# DNA Methylation Patterns in CD8^+^ T Cells Discern Psoriasis From Psoriatic Arthritis and Correlate With Cutaneous Disease Activity

**DOI:** 10.3389/fcell.2021.746145

**Published:** 2021-10-21

**Authors:** Amandine Charras, Jessica Garau, Sigrun R. Hofmann, Emil Carlsson, Cristina Cereda, Susanne Russ, Susanne Abraham, Christian M. Hedrich

**Affiliations:** ^1^Department of Women’s and Children’s Health, Institute of Life Course and Medical Sciences, University of Liverpool, Liverpool, United Kingdom; ^2^Genomic and Post-Genomic Unit, IRCCS Mondino Foundation, Pavia, Italy; ^3^Klinik und Poliklinik für Kinder- und Jugendmedizin, Universitätsklinikum Carl Gustav Carus, TU Dresden, Dresden, Germany; ^4^Klinik und Poliklinik für Dermatologie, Universitätsklinikum Carl Gustav Carus, TU Dresden, Dresden, Germany; ^5^Department of Paediatric Rheumatology, Alder Hey Children’s NHS Foundation Trust Hospital, Liverpool, United Kingdom

**Keywords:** psoriasis, psoriatic arthritis, CD8^+^ T cell, chromatin, DNA methylation, patient stratification

## Abstract

**Background:** Psoriasis is a T cell-mediated chronic autoimmune/inflammatory disease. While some patients experience disease limited to the skin (skin psoriasis), others develop joint involvement (psoriatic arthritis; PsA). In the absence of disease- and/or outcome-specific biomarkers, and as arthritis can precede skin manifestations, diagnostic and therapeutic delays are common and contribute to disease burden and damage accrual.

**Objective:** Altered epigenetic marks, including DNA methylation, contribute to effector T cell phenotypes and altered cytokine expression in autoimmune/inflammatory diseases. This project aimed at the identification of disease-/outcome-specific DNA methylation signatures in CD8^+^ T cells from patients with psoriasis and PsA as compared to healthy controls.

**Method:** Peripheral blood CD8^+^ T cells from nine healthy controls, 10 psoriasis, and seven PsA patients were collected to analyze DNA methylation marks using Illumina Human Methylation EPIC BeadChips (>850,000 CpGs per sample). Bioinformatic analysis was performed using R (*minfi, limma, ChAMP*, and *DMRcate* packages).

**Results:** DNA methylation profiles in CD8^+^ T cells differentiate healthy controls from psoriasis patients [397 Differentially Methylated Positions (DMPs); 9 Differentially Methylated Regions (DMRs) when ≥CpGs per DMR were considered; 2 DMRs for ≥10 CpGs]. Furthermore, patients with skin psoriasis can be discriminated from PsA patients [1,861 DMPs, 20 DMRs (≥5 CpGs per region), 4 DMRs (≥10 CpGs per region)]. Gene ontology (GO) analyses considering genes with ≥1 DMP in their promoter delivered methylation defects in skin psoriasis and PsA primarily affecting the BMP signaling pathway and endopeptidase regulator activity, respectively. GO analysis of genes associated with DMRs between skin psoriasis and PsA demonstrated an enrichment of GABAergic neuron and cortex neuron development pathways. Treatment with cytokine blockers associated with DNA methylation changes [2,372 DMPs; 1,907 DMPs within promoters, 7 DMRs (≥5 CpG per regions)] affecting transforming growth factor beta receptor and transmembrane receptor protein serine/threonine kinase signaling pathways. Lastly, a methylation score including TNF and IL-17 pathway associated DMPs inverse correlates with skin disease activity scores (PASI).

**Conclusion:** Patients with skin psoriasis exhibit DNA methylation patterns in CD8^+^ T cells that allow differentiation from PsA patients and healthy individuals, and reflect clinical activity of skin disease. Thus, DNA methylation profiling promises potential as diagnostic and prognostic tool to be used for molecular patient stratification toward individualized treatment.

## Introduction

Psoriasis is a chronic systemic autoimmune/inflammatory disease that primarily affects the skin (Schön, 2019). An estimated 11–30% of psoriasis patients develop arthritis and are therefore diagnosed with psoriatic arthritis (PsA; Gladman et al., 2002). Notably, arthritis can precede skin disease, sometimes by several years, thereby complicating correct diagnosis and delaying the introduction of effective treatment (Diani et al., 2015). While psoriasis can generally occur at any age, and both sexes are equally affected, two peak ages have been suggested in adolescence/young adulthood (16–22 years) and later in life (57–60 years) (Perera et al., 2012). Thus, psoriasis can impact on almost any life stage and causes significant burden and cost for the individual and society.

The pathophysiology of psoriasis, both skin psoriasis and PsA, is incompletely understood. Multiple factors have been discussed, including genetic susceptibility, environmental triggers in combination with skin barrier disruption, and general immune dysfunction (Ayala-Fontánez et al., 2016). Skin psoriasis and PsA are characterized by a pathological activation of the TNF/IL-23/IL-17 cytokine axis, contributing to an immunological imbalance favoring effector T cell differentiation and activation, as well as their accumulation in affected tissues (Di Cesare et al., 2009; Hawkes et al., 2018; Carvalho and Hedrich, 2021). T cells play a pivotal role in disease pathogenesis at all stages, including breach of tolerance and the initiation of a pro-inflammatory phenotypes, the establishment of chronic inflammation, damage and amplification of self-reactivity, the maintenance of clinically established inflammatory tissue lesions, and “spreading” of inflammatory disease from initial sites (e.g., the skin) to secondary sites of inflammation (e.g., extra-cutaneous manifestations) (Casciano et al., 2018).

Epigenetic modifications orchestrate gene expression by regulating the accessibility of DNA to transcription factors and the transcriptional complex. Alterations to the epigenome have been linked with the molecular pathophysiology of autoimmune/inflammatory conditions, and link genetic predisposition with disease expression (Surace and Hedrich, 2019). Because of its stability in biological samples (Gosselt et al., 2021), DNA methylation is the most widely studied epigenetic mark. It contributes to effector phenotypes and inflammatory cytokine expression in T cells from patients with various autoimmune/inflammatory conditions (Brandt et al., 2018). In psoriasis, CD4^+^, CD8^+^ as well as CD4^–^CD8^–^ (double negative) T cells are involved in the molecular pathogenesis following their stimulation by (auto-)antigens (Brandt et al., 2018; Carvalho and Hedrich, 2021).

This study aimed to identify disease- and outcome-specific DNA methylation signatures in CD8^+^ T cells from patients with skin psoriasis and PsA as compared to healthy controls.

## Materials and Methods

### Patient Cohort

Samples were collected from 26 individuals: nine healthy controls, 10 plaque-type skin psoriasis (Kim et al., 2017), and seven PsA patients (Singh et al., 2019). Demographic and clinical characteristics of study cohorts are summarized in Table 1. Psoriasis Area and Severity Index (PASI) scores (Feldman, 2005) were collected at all study visits from patients with skin psoriasis and PsA.

**TABLE 1:** Participant demographics.

Patient identification	Patient phenotype	Age	Gender	Ethnicity	PASI score	Systemic treatment
Control_1	Healthy control	27	Male	Caucasian	–	–
Control_2	Healthy control	25	Female	Caucasian	–	–
Control_3	Healthy control	35	Male	Caucasian	–	–
Control_4	Healthy control	25	Female	Caucasian	–	–
Control_5	Healthy control	51	Female	Caucasian	–	–
Control_6	Healthy control	42	Male	Caucasian	–	–
Control_7	Healthy control	26	Female	Caucasian	–	–
Control_8	Healthy control	34	Male	Caucasian	–	–
Control_9	Healthy control	25	Female	Caucasian	–	–
Psoriasis_1 before treatment	Psoriasis	33	Female	Caucasian	19.5	–
Psoriasis_1 after treatment	Psoriasis	33	Female	Caucasian	1.2	Anti-TNFα
Psoriasis_2 before treatment	Psoriasis	25	Male	Caucasian	16.3	–
Psoriasis _2 after treatment	Psoriasis	25	Male	Caucasian	3.1	Anti-IL17
Psoriasis_3 before treatment	Psoriasis	30	Male	Caucasian	20.7	–
Psoriasis_3 after treatment	Psoriasis	30	Male	Caucasian	1.8	Anti-IL17
Psoriasis_4 before treatment	Psoriasis	45	Male	Caucasian	21.8	–
Psoriasis_4 after treatment	Psoriasis	45	Male	Caucasian	4	Anti-IL17
Psoriasis_5	Psoriasis	51	Male	Caucasian	12.4	–
Psoriasis_6	Psoriasis	49	Male	Caucasian	14.9	–
Psoriasis_7	Psoriasis	37	Female	Caucasian	10.5	–
Psoriasis_8	Psoriasis	27	Female	Caucasian	10.2	–
Psoriasis_9	Psoriasis	51	Male	Caucasian	21.3	–
Psoriasis_10	Psoriasis	20	Male	Caucasian	21.5	–
PsA_1 before treatment	Psoriatic arthritis	30	Female	Caucasian	25.1	–
PsA_1 after treatment	Psoriatic arthritis	30	Female	Caucasian	11.2	Anti-TNFα
PsA_2	Psoriatic arthritis	40	Male	Caucasian	15	–
PsA_3	Psoriatic arthritis	70	Male	Caucasian	7.6	–
PsA_4	Psoriatic arthritis	49	Male	Caucasian	0.8	–
PsA_5	Psoriatic arthritis	51	Female	Caucasian	0.8	–
PsA_6	Psoriatic arthritis	81	Female	Caucasian	3.4	–
PsA_7	Psoriatic arthritis	62	Male	Caucasian	6	–

*PsA, Psoriatic arthritis.*

### CD8^+^ T Cell and Genomic DNA Isolation

Peripheral blood mononuclear cells (PBMCs) were *ex vivo* isolated from fresh blood samples obtained from patients and healthy controls using Biocoll (Merck) and Leucosep Tubes (Greiner, Bio-One), following standard protocols. Next, CD8^+^ T cells were separated from PBMCs using flow cytometry and FACS sorting with the following antibodies: Pacific Blue anti-CD4 (OKT4; BioLegend); FITC anti-CD3 (OKT3; BioLegend); PE anti-CCR7 (G043H7; BioLegend); APC anti-CD45RA (HI100; BioLegend), APC-Cy7 anti-CD8 (SK1; BioLegend). Cells were stained with aforementioned fluorophore-conjugated antibodies in 2% BSA, 1 mM EDTA/PBS on ice for 30 min, phenotyped, counted and collected on a FACSAria II cell sorter (Becton Dickinson) by gating on viable CD3^+^ and CD4^–^, CD8^hi^, CD8^lo^, or CD8^–^ T cell populations, as indicated (Supplementary Figure 1A). Datasets were analyzed using FlowJo software V10 (TreeStar).

Sorted CD8^+^ T cells were collected and stored at −80°C. Isolation and separation of genomic DNA from sorted cells was performed using ZR-Duet DNA/RNA MiniPrep kits (Zymo Research) according to manufacturer’s protocol (including DNAse step for RNA isolation). Genomic DNA quantity and quality was assessed using NanoDrop and Qubit (Thermo Fisher Scientific).

### DNA Methylation Profiling

The Illumina Infinium MethylationEPIC array BeadChip (850K) was carried out using Diagenode Epigenomic Services (Vienna, Austria, Cat No. G02090000). Genomic DNA samples from CD8^+^ T were sent for bisulfite conversion [EZ-96 DNA Methylation Kit (Zymo Research)] and DNA methylation profiling using the Illumina Human Methylation EPIC platform to analyze the methylation status of more than 850,000 CpGs per samples. This microarray covers ∼96% of CpG Islands and 99% of annotated RefSeq genes.

### Quality Control, Data Normalization and Statistical Analysis of Differentially Methylated Positions and Differentially Methylated Regions

Methylation profiles of CD8^+^ T cells were analyzed using R packages *Minfi* (Aryee et al., 2014) and *ChAMP* (Tian et al., 2017). Type 1 and type 2 probes were normalized using quantile normalization and BMIQ. The sex of donors was confirmed using the predictSex function of the *Minfi* package. The following probes were filtered out: (i) probes not passing the detection *p*-value cut-off of 0.01, (ii) probes with known SNPs, (iii) probes not in CpG context, and (iv) cross reactive probes [(McCartney et al., 2016; Pidsley et al., 2016) demonstrated that some probes map to multiple genomic sites and may therefore affect analysis]. Batch effects were corrected using the ComBat function of *ChAMP* package, which was derived from the *SVA* package (Johnson et al., 2007).

For downstream analysis and data visualization, M and Beta (β) values were generated: *M* values represent the base 2 log ratio of the intensities of the methylated and unmethylated probes; β values are the ratio of methylated probe intensities to the overall intensity. For statistical purposes, *M* values were used as β value distribution displays heteroscedasticity in low and high methylation ranges (Du et al., 2010). However, β values, which range from 0 (0% methylation) to 1 (100% methylation) were used for data visualization.

Differentially methylated positions (DMPs) between groups were determined using empirical Bayes’ moderated *t* test method, contained in the *limma* package (Ritchie et al., 2015). False discovery rates (FDR) <0.05 were used as significance threshold. Only probes with a difference in β values over 10% were kept for analysis (|Δβ| > 0.1).

Differentially methylated regions (DMRs) were identified using the *DMRcate* package (Peters et al., 2015). The matrix of *M* value (logit transformation of beta) is annotated with the relevant annotation information about the probes such as their genomic position, gene annotation, etc. The limma pipeline was used for differential methylation analysis and to calculate moderated t-statistics; the dmrcate() function was used to combine CpGs to extract DMRs with a β value cut-off of 10% and FDR < 0.05 and a minimum number of CpG of 5, 10, and 20.

### Gene Ontology

In a first step, gene enrichment analysis was performed for genes presenting at least one promoter DMP (TSS1500, TSS200, 5′UTR), followed by analysis based on DMPs in promoters and gene bodies. Gene Ontology (GO) analysis for biological processes, cellular components and molecular functions and KEGG pathway analysis (Kyoto Encyclopedia of Genes and Genomes)^1^ were performed using the R package *clusterProfiler* (Yu et al., 2012). Only significant GO terms and KEGG pathways are shown (Bonferroni corrected *p* < 0.05).

### Calculation of Methylation Scores (mDNA Scores)

To identify potential DMPs associating with disease activity before and after treatment, genes involved in TNF-α and IL-17 signaling pathways were identified through the WikiPathways database^2^ (Martens et al., 2021). We focused on these genes, as patients involved in this target identification step of the study were treated with either TNF or IL-17A directed biopharmaceutical agents. Methylation scores were calculated as previously suggested by Björk et al. (2020). Briefly, means (mean_HC_) and standard deviations (SD_HC_) for each DMP involved in TNF-α and IL-17 signaling pathways in the healthy control group were used to achieve standardized values (SVs) for each individual according to the formula: SV = (Value-Mean_HC_)/SD_HC_. Subsequently, SVs were summed up to total scores (Björk et al., 2020).

### Statistical Analysis

One-way ANOVA followed by Tukey’s *post hoc* test and Kruskal–Wallis followed by Dunn’s *post hoc* tests were used when comparing more than two groups (normality and homoscedasticity were tested prior to these tests). Shapiro–Wilk normality tests were performed to assess Gaussian distribution before testing statistical associations between two variables using Pearson’s correlation. Statistical tests were performed using GraphPad Prism (V.6.0, GraphPad).

## Results

### Study Cohort

We investigated differential methylation patterns of CD8^+^ T cells from patients with skin psoriasis (*N* = 10), PsA patients (*N* = 7), and healthy controls (*N* = 9). An imbalance existed in the distribution between women and men across sub-cohorts with more men in the skin psoriasis (70%) and PsA (57%) patient cohorts when compared to healthy controls (44%). Furthermore, the mean age of skin psoriasis (36.8 years) and PsA (54.7 years) patients was slightly higher when compared to healthy controls (32.2 years) (Table 1).

### Peripheral Blood Effector Memory CD8^+^ T Cells Are Elevated in Psoriatic Arthritis Patients

CD8^+^ T cells were FACS sorted and phenotyped (described in section “Materials and Methods”) to distinguish the proportion of naïve, Effector Memory (EM), Central Memory (CM), and Effector memory cells re-expressing CD45RA (EMRA) sub-population by flow cytometry (Supplementary Figure 1A). While we observed comparable proportions of CD8^+^ T cells across study cohorts (Supplementary Figure 1B), we detected significantly increased proportions of EM CD8^+^ T cells in PsA patients when compared to healthy controls (Supplementary Figure 1C).

### Differentially Methylated CpG Positions Separate Psoriasis Patients From Controls, and Skin Psoriasis From Psoriatic Arthritis

**FIGURE 1:**
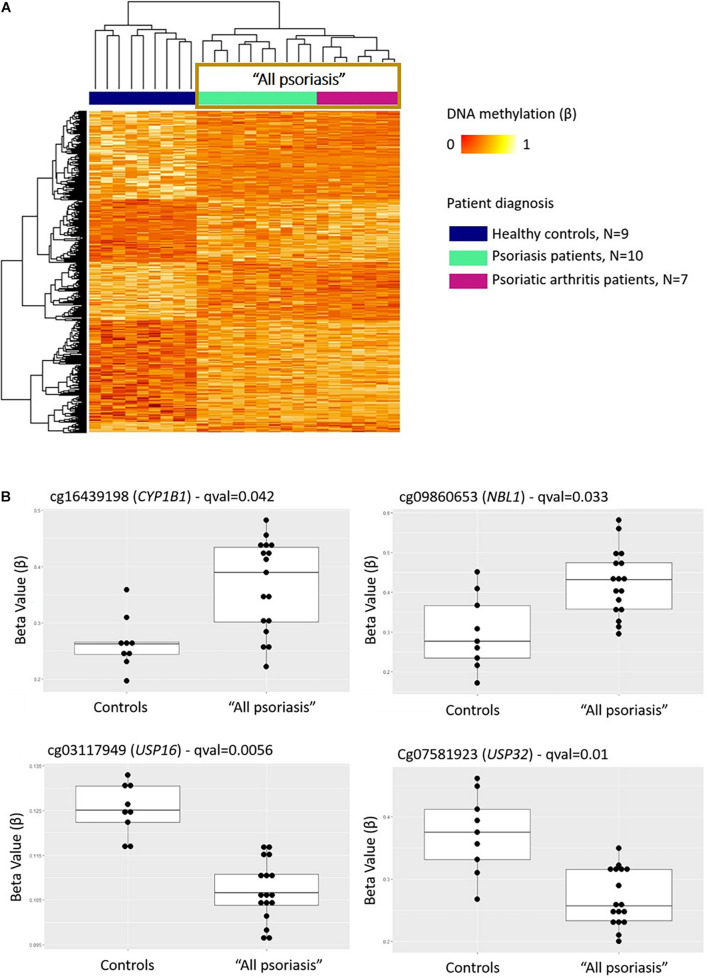
DNA methylation profiles differentiate CD8^+^ T cells from psoriasis patients and healthy individuals. **(A)** Heat map showing differentially methylated positions (DMP) between “all psoriasis” (combined psoriatic arthritis and psoriasis patients) patients and healthy controls (FDR < 0.05, |Δβ| > 0.1). Normalized DNA methylation levels are displayed on the right with red indicating reduced methylation and yellow indicating increased methylation levels. **(B)** Differences in beta values of selected CpG sites identified as DMPs across “all psoriasis” patients versus healthy controls. “All psoriasis,” Psoriasis and PsA patients combined.

Comparison between “all psoriasis” (combined skin psoriasis and PsA) patients versus healthy controls identified 397 differentially methylated positions (DMPs), including 195 hypermethylated and 202 hypomethylated CpGs (FDR < 0.05, |Δβ| > 0.1) (Table 2). Differential global DNA methylation patterns (Figure 1A) and detailed examples of differentially methylated CpG sites (top four candidates, Figure 1B) are displayed in Figure 1.

**TABLE 2:** Differentially methylated positions (DMPs) in CD8^+^ T cells from psoriasis patients and controls.

	DMPs			Corresponding genes			
Comparison	Hypermethylated	Hypomethylated	Total	Hypermethylated	Hypomethylated	Hyper and hypomethylated	Total
Controls vs. Psoriasis	110	224	334	73	140	1	212
Controls vs. PsA	957	1138	2095	602	644	24	1222
Psoriasis vs. PsA	987	874	1861	642	508	24	1126
“All psoriasis” vs. Controls	195	202	397	124	123	3	244
Before vs. after treatment	508	1864	2372	443	1460	59	1962

*DMPs were obtained between groups with criteria selection *q* value < 0.05 and |Δβ| ≥ 0.1 and genes concerned. PsA, Psoriatic arthritis; All psoriasis, Psoriasis and PsA patients combined.*

**FIGURE 2:**
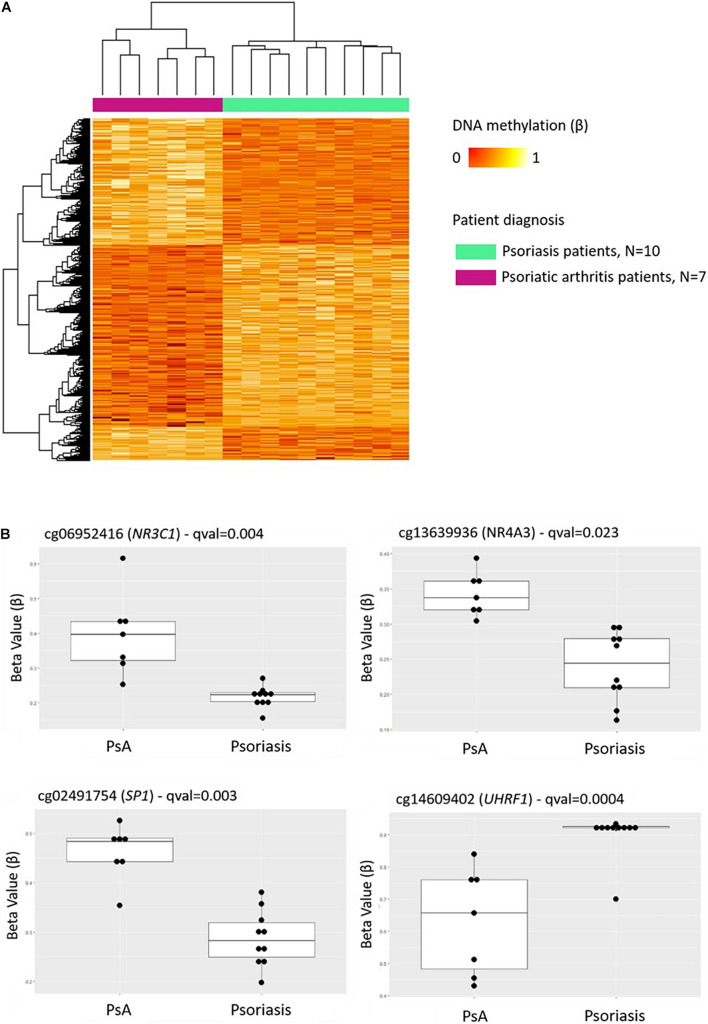
DNA methylation profiles differentiate CD8^+^ T cells from psoriatic arthritis and skin psoriasis patients. **(A)** Heat map showing differentially methylated positions (DMP) between psoriatic arthritis patients and psoriasis patients (FDR < 0.05, |Δβ| > 0.1). Normalized DNA methylation levels are displayed on the right with red indicating reduced methylation and yellow indicating increased methylation levels. **(B)** Differences in beta values of selected CpG sites identified as DMPs across psoriasis and psoriatic arthritis patients. PsA, Psoriatic arthritis patient.

Comparison of DNA methylation patterns in CD8^+^ T cells from psoriasis vs. PsA patients allowed the identification of 1,861 DMPs, including 987 hypermethylated and 874 hypomethylated CpGs (Table 2 and Figure 2A). Differential global DNA methylation patterns (Figure 2A) and detailed examples of differentially methylated CpG sites (top four candidates, Figure 2B) are displayed in Figure 2.

**FIGURE 3:**
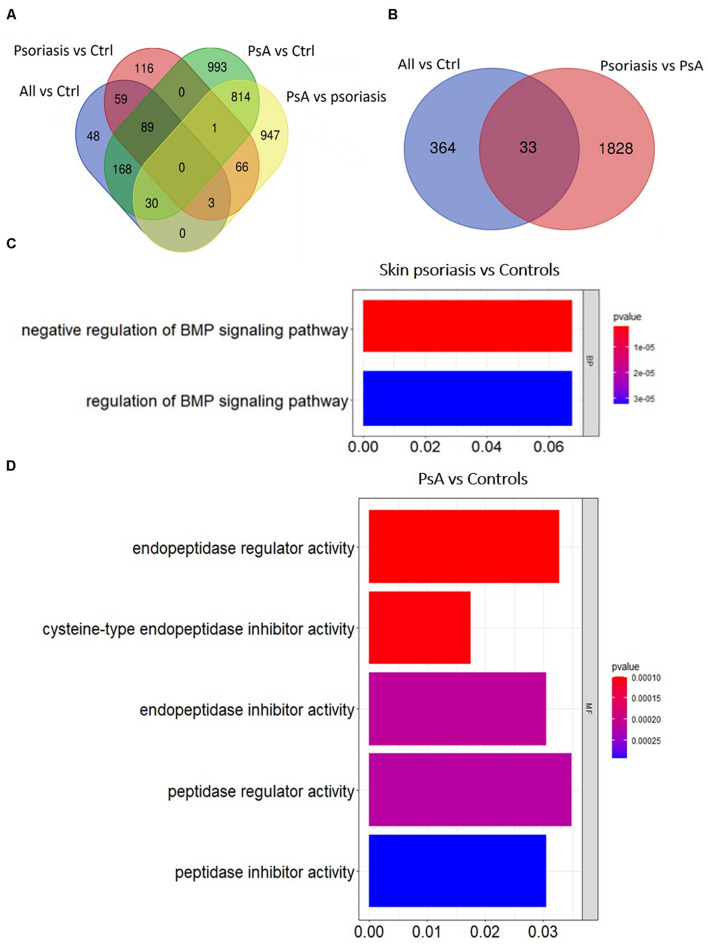
Shared and unique methylation patterns in CD8^+^ T cells from psoriasis patients and controls, and pathways affected. **(A)** Venn diagram showing overlapping and differentially methylated positions (DMP) found in psoriasis patients versus controls (Ctrl). Differences were tested between “All” (skin psoriasis and psoriatic arthritis combined) and Ctrl (healthy control), controls versus skin psoriasis patients, controls versus psoriatic arthritis (PsA) patients, and skin psoriasis versus PsA patients **(A)**. **(B)** Contrasts were assessed only between “All” and Ctrl and skin psoriasis versus PsA patients. **(C,D)** The bar plots show the results of the Gene Ontology (GO) analysis of genes which presented at least on DMP in their promoter. Only significantly enriched terms for Biological Process (BP), Cellular Component (CC) and Molecular Function (MF) are represented (*P* < 0.05), in “skin psoriasis” versus Controls **(C)** and PsA versus Controls **(D)**.

Overall numbers of DMPs identified across all aforementioned analyses are displayed in Figure 3. No DMPs (Figure 3A) were common to all four sub-analyses; 33 DMPs were shared between “all psoriasis” patients versus controls and psoriasis versus PsA analyses (Figure 3B), accounting for 8% of DMPs identified in “all psoriasis” patient versus healthy controls, and almost 2% of DMPs identified in psoriasis versus PsA analyses. Results illustrate unique methylation profiles across patient and control cohorts.

### Genomic Distribution of Differentially Methylated Positions Across Study Cohorts

Considering DNA methylation patterns in CD8^+^ T cells from “all psoriasis” patients versus healthy controls, DMPs were equally distributed among promoter regions (31.9%), gene bodies (33.9%), and intergenic regions (32.4%) (Table 3). Analyzing DNA methylation in the context of CpG density, the majority of DMPs were found in the “open sea” (53.5%), followed by CpG islands (20.4%).

**TABLE 3:**
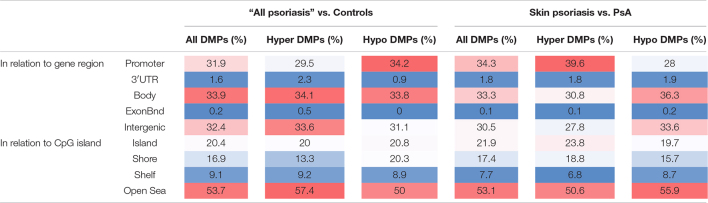
Functional genomic distribution of DMPs in CD8^+^ T cells from psoriasis, psoriatic arthritis patients, and controls.

*Functional genomic distribution of differentially methylated positions (All DMPs), hypermethylated (hyper DMPs), and hypomethylated (hypo DMPs) DMPs is displayed between “All patients” (psoriasis and psoriatic arthritis combined) versus controls (left) and psoriasis versus psoriatic arthritis patients (PsA, right). Analyses were performed in relation to gene region [promoter, 3′UTR, gene body, exon boundaries (Bnd), and intergenic regions] and CpG island context (Island, shore, shelf, open sea). Values are percentages and color scale indicate a high percentage (red) or low percentage (blue) of the DMPs in each functionally annotated region.*

Examination of DMP distribution between psoriasis patient sub-cohorts (skin psoriasis vs. PsA) revealed a similar distribution of DMPs between promoters (33.3%), gene bodies (34.3%), and intergenic regions (30.5%) (Table 3). As above, analysis of DMPs localization in relation to CpG density delivered a general predominance of DMPs in the “open sea” (53.1%) (Table 3). Notably, across all aforementioned comparisons, DMPs to almost equal extends exhibited hyper- or hypo-methylation between groups.

### Biological Pathway Analysis Separates Patients From Controls

Gene ontology (GO) analyses were performed to predict biological pathways affected by differential DNA methylation. First, analyses were limited to genes with at least one DMP in their promoter region. Analysis of 125 genes that included 154 DMPs (85 hypo and 69 hypermethylated) did not allow the identification of specific pathways between “all psoriasis” versus healthy controls. However, a total of 140 DMPs (98 hypo- and 42 hypermethylated CpGs) annotated to 111 unique genes were identified in skin psoriasis patients as compared to healthy controls. Gene ontology analysis of differentially methylated genes highlighted negative (*p* = 1.82 × 10^–6^) and positive (*p* = 3.22 × 10^–5^) regulation of the bone morphogenic protein (BMP) signaling pathway (Figure 3C). An even higher number of DMPs were identified between PsA patients and healthy controls. A total of 872 DMPs (482 hypo- and 449 hypermethylated CpGs) were identified and mapped to 667 unique genes linked to “endopeptidase regulator activity” (*p* = 9.92 × 10^–5^), “cysteine-type endopeptidase inhibitor activity” (*p* = 1.03 × 10^–4^), and “endopeptidase inhibitor activity” (*p* = 2.09 × 10^–4^) (Figure 3D).

Investigating variable DNA methylation in CD8^+^ T cells from patients with skin psoriasis versus PsA, we discovered 813 DMPs (524 hypo- and 289 hypermethylated CpGs) that were uniquely annotated to 613 genes. Though a high number of DMPs were identified, no significantly enriched pathways have been found applying GO analysis.

Next, GO analyses were performed considering genes presenting DMPs in both promoters and gene bodies. Analysis of 516 DMPs uniquely associated to 279 genes allowed the identification of “synapse organization” (*p* = 3.23 × 10^–5^) and “cell junction assembly” (*p* = 3.31 × 10^–5^) as enriched biological processes between healthy controls and “all psoriasis” patients (Supplementary Figure 2A). However, GO analysis did not highlight pathways differentially affected between skin psoriasis patients and healthy controls, although 247 genes presented at least one DMP in their promoter and gene body. A meaningful number of DMPs (2,746) was observed between PsA patients and healthy controls, which mapped to 1,379 unique genes linked to a variety of biological processes, cellular components and molecular functions. Among the most significant enriched pathways there were: “synapse organization” (*p* = 1.90 × 10^–8^), “cell junction assembly” (*p* = 2.34 × 10^–6^), and “cell-cell adhesion via plasma-membrane adhesion molecules” (*p* = 1.99 × 10^–5^) (Supplementary Figure 2C and Supplementary Table 1).

Finally, investigating DMPs in CD8^+^ T cells from patients with skin psoriasis versus PsA, a total of 2,457 DMPs were identified across 1,259 genes. DMPs corresponded also to a high number of biological processes associated, including “pattern specification process” (*p* = 9.36 × 10^–8^), “cartilage development” (*p* = 9.34 × 10^–6^), and “histone modification” (*p* = 1.74 × 10^–5^) (Supplementary Figure 2B and Supplementary Table 2).

### Identification of Differentially Methylated Regions

To identify larger differentially methylated genomic regions (DMRs), step-wise analysis including a minimum of 5, 10, or 20 CpGs per region was performed (Supplementary Tables 3–5).

**FIGURE 4:**
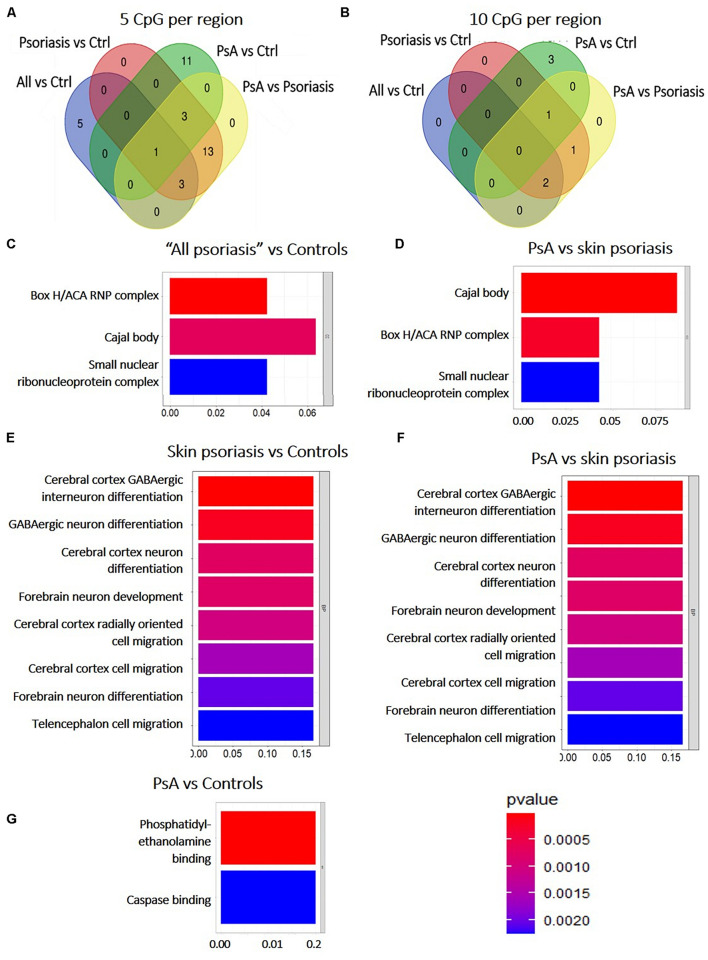
Shared and unique Differentially Methylated Regions (DMRs) in CD8^+^ T cells from psoriasis patients and controls, and pathways affected. **(A)** Venn diagram showing overlapping and differentially methylated regions (DMRs) with ≥5 CpGs **(A)** and ≥10 CpGs **(B)** per regions, in “all patients” (all, including psoriasis and psoriatic arthritis patients) versus controls (ctrl), controls versus psoriasis, controls versus psoriatic arthritis (PsA), and psoriasis versus psoriatic arthritis. **(C–G)** The bar plot shows the results of the Gene Ontology (GO) analysis of genes associated with DMRs with ≥5 CpGs per region between “all patients” versus controls **(C)** and PsA versus skin psoriasis **(D)**; and ≥10 CpGs for PsA versus skin psoriasis **(F)**, skin psoriasis versus controls **(E)**, and PsA versus controls **(G)**. Only significantly enriched terms for Biological Process (BP) and Cellular Component (CC) are represented (*P* < 0.05).

Among DMRs identified between “all psoriasis” patients versus healthy controls, skin psoriasis patients versus healthy controls, PsA patients versus healthy controls, and skin psoriasis versus PsA patients with ≥5 CpGs, only 1 DMR within the *ZNF714* gene was common to all analyses (Figure 4A). No DMRs were common to all analyses when DMRs with a minimum of 10 CpG per region were considered (Figure 4B). One DMR with ≥20 CpGs was common to all analyses in the *LHX6* gene (Supplementary Table 5).

Subsequently, GO analysis was performed to predict biological pathways affected by DMRs. A total of 20 DMRs with ≥5 CpGs associated to 78 genes differentiated skin psoriasis patients from healthy controls, and affected genes associated with the box H/ACA RNP complex (*p* = 0.007), Cajal body (*p* = 0.01), and the small nucleolar ribonucleoprotein complex (*p* = 0.02) (GO analysis, Figure 4C). Similarly, 20 DMRs affecting 78 genes differentiated PsA and skin psoriasis patients, and affected Cajal body, box H/ACA RNP complex, and small nucleolar ribonucleoprotein complex (*p* = 0.001, *p* = 0.004, *p* = 0.02, respectively) signaling pathways (GO analysis, Figure 4D).

Focusing on DMRs with ≥10 CpGs, a total of 4 DMRs affecting 11 genes differentiated skin psoriasis patients from healthy controls, and affected cerebral cortex GABAergic interneuron differentiation (*p* = 0.04), GABAergic, cerebral cortex and forebrain neuron differentiation, forebrain neuron development and cerebral cortex radially oriented and telencephalon cell migration (all *p* = 0.04) pathways (GO analysis, Figure 4E). Comparing PsA patients and healthy controls, 4 DMRs affecting 17 genes were identified and involved phosphatidylethanolamine and caspase binding (both *p* = 0.03) (Figure 4F).

Lastly, 4 DMRS affecting 11 genes were identified when comparing PsA and skin psoriasis. Notably, GO analysis highlighted the same signaling pathways previously identified when comparing skin psoriasis to healthy control patients (Figure 4G).

### Therapeutic Cytokine Blockade Impacts DNA Methylation in Psoriasis Patients

To investigate the impact of treatment on DNA methylation patterns in CD8^+^ T cells from psoriasis patients, DMP analysis was conducted before and after treatment initiation with cytokine blocking strategies (anti-TNF or anti-IL-17A treatment).

A comparable proportion of CD8^+^ T cells was observed in patients before and after treatment. While no differences between the proportions of naïve, EM and CM CD8^+^ T cells was observed, treatment with cytokine blockers associated with a larger proportion of effector memory re-expressing CD45RA CD8^+^ T cells (EMRA) (Supplementary Figure 3).

**FIGURE 5:**
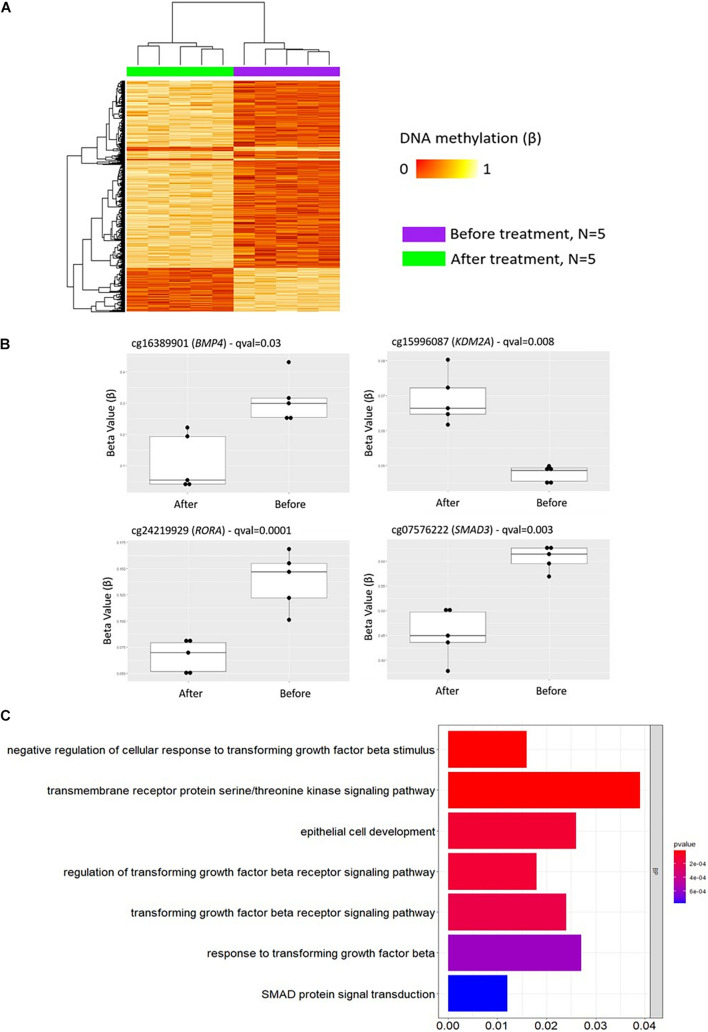
DNA methylation profiles in CD8^+^ T cells from patients with psoriasis change in response to treatment. **(A)** Heat map showing differentially methylated positions (DMP) in CD8^+^ T cells from psoriasis (*N* = 4 psoriasis and *N* = 1 psoriatic arthritis) before and after treatment (FDR < 0.05, |Δβ| > 0.1) with TNF inhibitors or inactivating IL-17A antibodies. Normalized DNA methylation levels are shown on the right red indicating reduced methylation and yellow indicating increased methylation levels. **(B)** Differences in beta values of selected CpG sites identified as DMP comparing status “before” versus “after” treatment initiation. **(C)** The bar plot shows the results of the Gene Ontology (GO) analysis of genes which presented at least on DMP in their promoter. Only significantly enriched terms for Biological process (BP) are represented (*P* < 0.05).

A total of 2,372 DMPs (508 hyper- and 1,864 hypomethylated CpGs) were identified in relation to treatment initiation (FDR < 0.05, |Δβ| > 0.1) (Table 2). Differential global DNA methylation patterns (Figure 5A) and detailed examples of differentially methylated CpG sites (top four candidates, Figure 5B) are displayed in Figure 2. Generally, DMPs were enriched in promoters (57%) and CpG islands (29.8%, Table 4), with a mild predominance of hypermethylated positions in gene promoters (69.9%) and shore regions (26.2%), and more hypomethylation within CpG Island (72.5%).

**TABLE 4:**
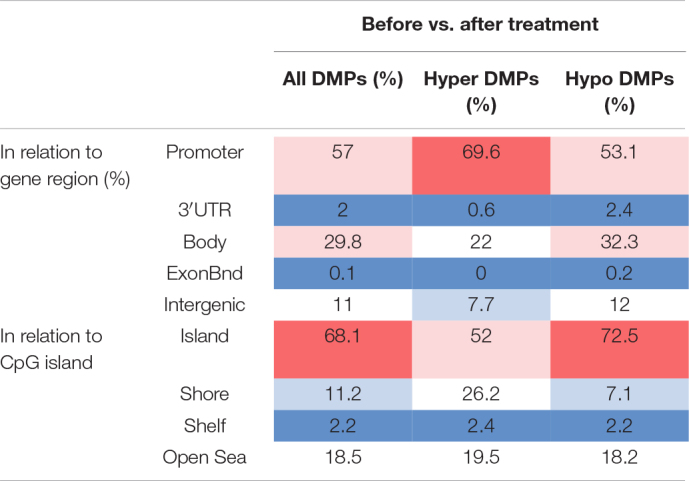
Functional genomic distribution of DMPs in CD8^+^ T cells in response to treatment.

*Functional genomic distribution of differentially methylated positions (All DMPs), hypermethylated (hyper DMPs), and hypomethylated (hypo DMPs) DMPs is displayed between psoriasis patients “before” and “after” treatment initiation with TNF inhibitors or inactivating IL-17A antibodies. Analyses were performed in relation to gene region (promoter, 3′UTR, gene body, exon boundaries, and intergenic regions) and CpG island context (Island, shore, shelf, open sea). Values are percentages and color scale indicate a high percentage (red) or low percentage (blue) of the DMPs in each functionally annotated region.*

As in GO analyses conducted above, initially only DMPs within promoter regions alone (TSS1500, TSS200, 5′UTR) were considered. Comparing DNA methylation profiles of “all psoriasis” patients before and after treatment with anti-TNF or anti-IL17A directed agents, we identified 1,907 DMPs (1,359 hypo- and 548 hypermethylated) annotated to 1,380 genes. GO pathway analysis revealed an enrichment of genes involved in a total of 95 biological processes (Supplementary Table 6). In Figure 5C, signaling pathways associated with psoriasis (skin psoriasis or PsA) are displayed and include “negative regulation of cellular response to transforming growth factor beta stimulus” (*p* = 1.54 × 10^–5^), “transmembrane receptor protein serine/threonine kinase signaling pathway” (*p* = 1.69 × 10^–5^), “epithelial cell development” (*p* = 1.14 × 10^–4^), and “SMAD protein signal transduction” (*p* = 7.72 × 10^–4^). Additionally, GO analyses considering genes with at least one DMP in their promoter and gene body were performed, and delivered 3,883 DMPs uniquely associated to 2,055 genes which linked to multiple pathways, including “embryonic skeletal system development” (*p* = 1.32 × 10^–8^), “histone deacetylase complex” (*p* = 2.75 × 10^–5^) and “transcription regulator complex” (*p* = 1.69 × 10^–5^) (Supplementary Figure 4 and Supplementary Table 7).

Considering wider DMRs, differentially methylated regions were identified in patients before and after treatment only with a minimum number of CpG of 5 (7 DMRs, Table 5).

**TABLE 5:** DMRs in CD8^+^ T cells from patient before versus after treatment with a minimum number of 5 CpG per region.

	Seq. names	Start	End	Width	Strand	No. CpGs	min_smoothed_fdr	Stouffer	HMFDR	Fisher	Max diff	Mean diff	Overlapping genes
1	chr7	4829256	4831038	1783	*	7	0	2.29E-06	7.79E-11	1.60E-10	0.743623188	0.12051242	AP5Z1
2	chr1	1.6E + 08	1.6E + 08	1366	*	6	0	1.54E-07	1.60E-09	6.86E-10	0.616346078	0.113332116	snoU13, Y_RNA, SCARNA16, SNORD112, SNORA63, U3, SNORA51, SNORA25, SNORD59, SCARNA20, SNORA67, U6, SNORA70, SNORA77, SNORA26, SNORA72, U8, SNORA31, SNORA40, CCDC19, ACA64, SNORD78, snoU109, SNORD60, SNORD116
3	chr17	36622522	36623419	898	*	5	0	0.039037453	1.18E-10	1.33E-07	0.684386166	0.142011824	SNORA69, ARHGAP23
4	chr1	33740732	33742369	1638	*	6	0	0.000709788	6.53E-10	1.38E-07	0.670186925	0.11635636	snoU13, Y_RNA, SCARNA16, SCARNA21, U1, SCARNA17, SCARNA18, SCARNA24, ZNF362, SNORD112, SNORA62, SNORA63, SNORD46, SNORA2, SNORD81, U3, SNORA51, SNORA25, SCARNA20, SNORA67, U6, SNORA70, SNORA77, SNORA26, U8, SCARNA11, SNORA31, SNORA42, SNORA40, SNORD64, ACA64, snoU109, SNORD60
5	chr11	1.34E + 08	1.34E + 08	1325	*	5	0	0.003851736	3.83E-09	1.58E-07	0.542962362	0.117641489	B3GAT1
6	chr10	1451166	1451609	444	*	6	0	0.080595592	6.16E-09	3.96E-06	0.60478433	0.105749797	ADARB2
7	chrX	1.35E + 08	1.35E + 08	1187	*	7	1.43E-113	0.393049518	8.90E-06	0.002136723	−0.806406846	−0.133938921	MMGT1

* is considered to belong to both strands.

### DNA Methylation Correlates With Skin Disease Activity

**FIGURE 6:**
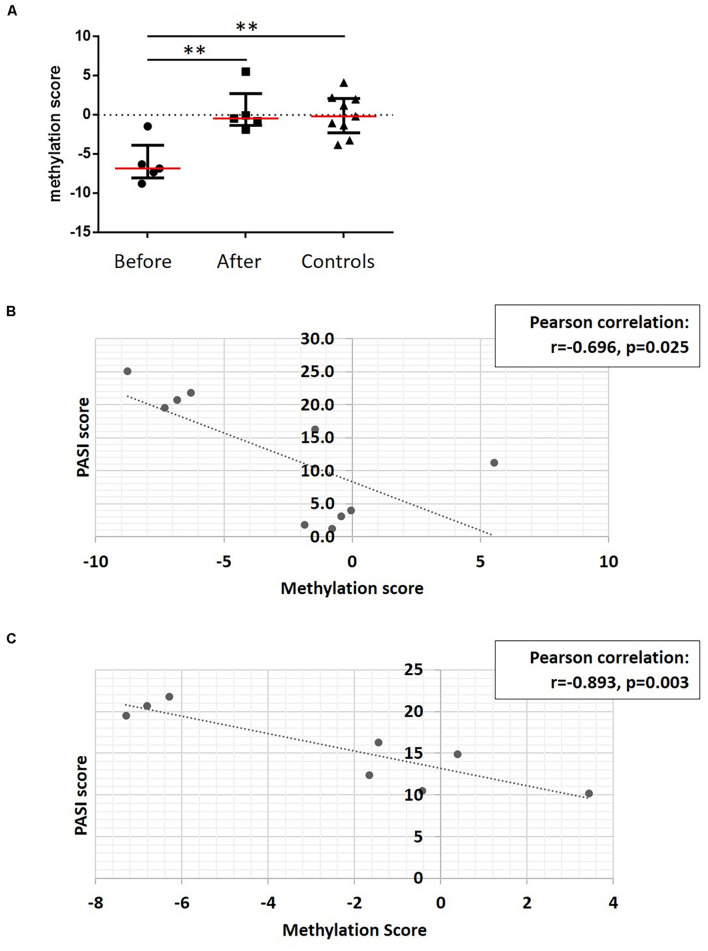
A DNA methylation score associates with disease activity. **(A)** DNA methylation scores were calculated in patients before treatment, and after treatment and healthy control. **p ≤ 0.01, Dunn’s multiple comparisons test. Median (red) and interquartile range are represented in this scatter plot. **(B)** Correlation analysis between methylation scores and PASI scores in patient cohort before and after treatment. After assessing Gaussian distribution, Pearson was used to measure the correlation. **(C)** Correlation analysis between methylation scores and PASI scores in the psoriasis patient cohort. After assessing Gaussian distribution, Pearson was used to measure the correlation. Dotted lines indicate a trendline.

To develop a tool to measure skin psoriasis disease activity, DNA methylation changes in response to treatment were investigated and correlated with skin disease activity as assessed by PASI scores. As anti-TNF and anti-IL-17A treatments were applied and both cytokines play a key role in the pathophysiology of psoriasis (Blauvelt and Chiricozzi, 2018), the search was focused on these two biological pathways. Genes involved in TNF-α and IL-17 signaling were identified using the WikiPathways database (see text footnote 2) (Martens et al., 2021). Following this, 10 DMPs previously identified and associated with these genes were considered. Beta values were collected, and exclusively DMPs for which β values strongly correlated with skin disease activity/PASI scores (correlation coefficient above |0.8|) were used to calculate methylation scores (Björk et al., 2020). Notably, methylation scores increased in response to treatment becoming comparable to methylation scores in healthy individuals (Figure 6A). Indeed, methylation scores inversely correlated with PASI scores (*r* = −0.696 and *p* = 0.025, Figure 6B).

To assess whether methylation scores correlate with skin disease activity of psoriasis patients independent of treatment initiation, they were tested in the sub-cohort of patients with skin psoriasis. Indeed, a strong negative correlation was observed between PASI and methylation scores (*r* = −0.893, *p* = 0.003, Figure 6C).

## Discussion

We, for the first time, show that patients with psoriasis exhibit DNA methylation patterns in CD8^+^ T cells that allow differentiation from healthy controls. Furthermore, we demonstrate that DNA methylation marks in CD8^+^ T cells from PsA patients and patients with skin psoriasis are distinct, suggesting DNA methylation screening as a promising candidate in the search for diagnostic and predictive biomarkers. This is of high potential value for translation into clinical care, as up to 25–50% of patients with PsA develop arthritis sometimes years before the onset of skin involvement (Busse and Liao, 2010). Furthermore, as joint disease in PsA is destructive and results in significant damage and disability, early recognition, delay of its onset or even prevention may be possible through identification of individuals at risk (Scher et al., 2019). However, at this stage, only samples from patients with current skin psoriasis or PsA were included in this study, and prospective monitoring of patients some of who will develop joint involvement is necessary to reliably answer the question of whether DNA methylation patterns change prior to disease progression from initial skin disease to PsA.

In addition to potential clinical applications as disease biomarkers, DNA methylation patterns also offer insights into the molecular pathophysiology of autoimmune/inflammatory conditions (Hedrich and Tsokos, 2011; Charras and Hedrich, 2019; Surace and Hedrich, 2019). Identification of molecular pathways affected may therefore offer insights into the pathogenesis of disease and direct future experimental work. Thus, functional gene ontology analyses were conducted examining genes containing at least one DMP in their promoter region. Interestingly, we only observed significant pathway associations between skin psoriasis or PsA patients and controls, but not between psoriasis sub-cohorts. This likely highlights skin psoriasis and PsA representing clinical phenotypes within the spectrum of “psoriatic disease” rather than distinct conditions (Sakkas and Bogdanos, 2017). Differences between all psoriasis patients and controls, but not between disease sub-types, may also reflect the importance of immunological interplay between immune cell populations beyond CD8^+^ T cells (Teschendorff and Zheng, 2017).

Comparing skin psoriasis and healthy controls, GO analysis revealed DMP enrichment affecting distinct pathways, including negative and positive regulation of bone morphogenic protein (BMP) signaling. The BMP signaling pathway is an important regulator of epidermal homeostasis, hair follicle growth, melanogenesis and has previously been linked with the pathobiology of psoriasis (Botchkarev, 2003). Notably, Sconocchia et al. (2021) recently suggested functional links between BMP signaling and regulatory CD4^+^ T cell (Treg) accumulation in psoriatic skin lesions. This is further supported by reports of dysregulated BMP-4 expression in psoriatic skin lesions that recover after 16 weeks of treatment with the TNF inhibitor adalimumab (Di Costanzo et al., 2019). Lastly, another BMP family member, BMP7 was suggested to locally promote Treg differentiation (Borek et al., 2020).

When comparing DMPs in CD8^+^ T cells from PsA versus healthy controls, “endopeptidase regulator activity,” “cysteine-type endopeptidase inhibitor activity,” and “endopeptidase inhibitor activity” were among candidate pathways identified. Cysteine proteases are a group of endopeptidases that contain a Cys-His-Asn triad at the active site (Verma et al., 2016). Notably, cathepsin K plays a crucial role in bone remodeling and is predominantly expressed in osteoclasts (Rengel et al., 2007). Cathepsin K plays a key role in the development of psoriasis-like lesions in mouse models of psoriasis by affecting Th17 polarization (Hirai et al., 2013). Notably, PsA patients exhibit increased cathepsin K serum levels when compared with psoriasis patients and controls, which negatively correlates with calcitonin serum levels (Cubillos et al., 2016). Calcitonin is a hormonal calcium sensor that reduces bone resorption and remodeling by osteoclasts (Xie et al., 2020). Furthermore, Cysteine endopeptidases are involved in extra-cellular-matrix remodeling and their dysregulation has been linked to various diseases, including fibrosis and osteoarthritis (Bonnans et al., 2014).

The inclusion of DMPs in both promoters and gene bodies allowed the identification of an even higher number of pathways affected by DNA methylation defects, some of which have been linked with disease pathophysiology and clinical outcomes. Comparing all sub-types of psoriasis with healthy controls and PsA patients with healthy controls, the involvement of “cell junction assembly” and “cell-cell adhesion via plasma-membrane adhesion molecules” were highlighted. Currently available literature suggests that alterations of tight junctions proteins play a role in the pathophysiology of psoriasis (Kirschner et al., 2009), and antigens related to endothelial cell activation are important for the maintenance of cell aggregates in inflamed skin and infiltration of leukocytes (Lee et al., 1994; Veale et al., 1995). Notably, “cartilage development” was among the most relevant pathways observed when comparing PsA and skin psoriasis patients. Cartilage damage is one of the main characterizing features of PsA through disease stages, and proteoglycan loss is strictly associated to periarticular inflammation and synovitis (Abrar et al., 2020).

Differentially methylated regions (DMRs) are composed of multiple consecutive CpG sites that can regulate cellular functions including cell differentiation, proliferation, and aging (Reik et al., 2001; Bjornsson et al., 2008; Bock et al., 2008). Inter-individual variability in these processes is associated with differential methylation patterns in DMRs of multiple genetic loci (Lee et al., 1994; Bonnans et al., 2014). Moreover, DMRs are tissue specific and associate with disease state and may reflect autoimmune/inflammatory disease stages (Eckhardt et al., 2006). As coordinated DNA methylation changes in wider genomic regions are more likely to have downstream biological effects and implications for diseases development and progression than methylation changes in a single CpG, we analyzed DMRs across study sub-cohorts (Hotta et al., 2018; Spindola et al., 2019). Indeed, findings from DMP analyses were largely confirmed investigating wider DMRs, and additionally allowed differentiation between skin psoriasis vs. PsA in subsequent GO analyses. DMRs analysis highlighted an enrichment of pathways linked to cellular components between all psoriasis patients and healthy controls, including the Small nuclear ribonucleoprotein (snRNP) complex, for which a class of autoantigens known as RNA-associated molecules and autoantibodies recognizing snRNPs has been described in a variety of autoimmune/inflammatory diseases, including systemic lupus erythematosus (SLE), systemic sclerosis and mixed connective tissue disease (Kattah et al., 2010). Moreover, Cajal bodies are involved in the biogenesis of snRNPs and autoantibodies against them have been observed in SLE (Vázquez-Talavera et al., 2004).

One of the targets identified to distinguishing PsA and psoriasis from healthy controls, GABAergic neuron and cortex neuron development pathways, may appear surprising at first. However, increased GABA ligand and the GABA_A_ receptor expression has been previously reported in psoriatic skin lesions (Nigam et al., 2010). Indeed, the sensory nervous system may alter immune functions in the skin, thereby contributing to inflammatory disease (Ayasse et al., 2020). A cross-talk between the immune and the nervous system through neurotransmitters has recently been proposed in psoriasis (Chen et al., 2020). In this context, GABA may have immunoregulatory potential as it stimulates the expression of FoxP3, a transcription factor essential for regulatory T cell differentiation and the expression of immune regulatory cytokines IL-10 and TGF-β, the T cell regulatory co-receptor CTLA4, and the myeloid regulatory membrane glycoprotein SIRP-α (Bajić et al., 2020). Furthermore, neurotransmitters may affect the composition of the gut microbiome, a recently appreciated factor in psoriasis (Chen et al., 2020). Lastly, decreased GABA serum levels were noted in a majority of psoriasis patients with psycho-emotional disorders, a common comorbidity affecting psoriasis patients (Matiushenko et al., 2020). However, in the here presented study, no information on emotional health was collected.

Measuring disease activity is a challenge in systemic autoimmune/inflammatory disease, including psoriasis (Ballestar et al., 2020; Calle-Fabregat et al., 2020; Carvalho and Hedrich, 2021). Clinical scores, including PASI, are available but limited by their time-consuming and inter observer variability, especial in less experienced/specialized institutions (Fink et al., 2018). To develop tools to reliably and reproducibly monitor treatment response and gain additional insights into molecular mechanisms on inflammation in psoriasis, we investigated whether cytokine blockade (anti-TNF or IL-17A directed) and induction of clinical remission impact on DNA methylation patterns in CD8^+^ T cells from psoriasis patients. Indeed, in response to treatment, we identified DMPs previously associated with epithelial cell development, TGF-β, and SMAD pathways. SMAD is a pivotal intracellular effector for TGF-β and BMP signaling (Dituri et al., 2019), both involved in the pathophysiology of psoriasis (Borek et al., 2020; Sconocchia et al., 2021).

Provided treatment-associated changes to methylation marks, we wondered whether the calculation of targeted methylation scores correlate with skin disease activity (PASI). We focused our efforts on genes involved in TNF and IL-17 signaling, as both pathways have previously been linked with the molecular pathophysiology of psoriasis and are targeted by (also here applied) available treatment options (Sakkas and Bogdanos, 2017). Indeed, methylation scores based on the formula suggested by Björk et al. (2020), discriminated between patients with disease activity versus the achievement of remission. Furthermore, methylation scores were comparable between healthy controls and psoriasis patients after treatment initiation. To assess whether these methylation scores may be used to assess inflammatory activity of skin disease in an unbiased manner, we tested their association with PASI scores in the sub-cohort of skin psoriasis patients prior to the initiation of systemic treatment, and identified close inverse correlation between DNA methylation and PASI scores. While this requires to be confirmed in larger independent cohorts, including longitudinal follow-up of individuals, this promises potential for the use as prognostic biomarker.

Differential methylation status of CD8^+^ T cells may be affected by differences in subset distribution across disease groups. Indeed, PsA patients included in this study exhibited higher proportions of EM CD8^+^ T cells when compared to healthy controls. EM CD8^+^ T cells are memory cells that have lost the constitutive expression of CCR7, heterogeneously express CD62L, and express chemokine receptors and adhesion molecules that are required for homing to inflamed tissues (Sallusto et al., 2004). Their increased proportion in PsA patients is in agreement with previous observations in blood and synovial fluid of PsA patients and their association with systemic inflammation (Diani et al., 2019). Furthermore, differential DNA methylation in response to treatment initiation also associated with changes in CD8^+^ T cell subset distribution. We observed a higher proportion of CD45RA re-expressing EMRA CD8^+^ T cells in response to treatment with cytokines blocking therapies. This relatively recently described memory T cells subset can express a variety of pro-inflammatory cytokines. Previous reports suggest that their increase may be the result of persistent exposure to pathogens, reactivation of latent viruses, and an increased levels of oxidative stress (Philippe et al., 2019). In the context of our study, the cause and effects of their increase in psoriasis patients after treatment initiation remains unclear and requires future studies.

While the work presented here delivers new insights into the molecular pathophysiology of skin psoriasis and PsA, and suggests the use of DNA methylation mapping as a diagnostic and/or prognostic biomarker, it has limitations. Sample size is limited because of the relative rarity of PsA, and findings require to be confirmed in larger independent cohorts. Furthermore, it remains unclear whether some of the differences in DNA methylation marks between disease sub-cohorts are (at least partially) caused by variable effector CD8^+^ T cell distribution. This question will be addressed in future studies using single-cell DNA methylation screening. Lastly, the current work does not allow the assessment of DNA methylation impacting on gene transcription as RNA sequencing was not performed.

## Conclusion

Disease and subtype-specific DNA methylation patterns in CD8^+^ T cells from psoriasis patients suggest that, similarly to what is already part of routine care in some cancers, DNA methylation profiling may allow for patient stratification toward individualized treatment, risk assessment and care. Targeted methylation scores closely correlating with skin disease activity (PASI) may represent a promising tool for monitoring disease activity and treatment response. While findings are encouraging, they require to be prospectively and independently confirmed in larger cohorts.

## Data Availability Statement

The data presented in the study are deposited in the GEO repository, accession number: GSE184500.

## Ethics Statement

The studies involving human participants were reviewed and approved by the Faculty of Medicine Carl Gustav Carus, TU Dresden, Dresden, Germany. The patients/participants provided their written informed consent to participate in this study.

## Author Contributions

AC and JG performed the DNA methylation, GO analysis, and wrote the first draft of the manuscript. SH and SR isolated immune cells and DNA. SH, SR, and EC analyzed flow cytometric datasets. SA consented patients and collected clinical data and biospecimen. CH oversaw all experimental and analytic steps and wrote the first draft of the manuscript. CH, SH, and SA planned the study. AC, JG, EC, CC, SR, SA, and CH were involved in individual or all steps of data analysis and overall data interpretation. All authors read, commented and agreed to the final version of the manuscript and the authors’ list.

## Conflict of Interest

The authors declare that the research was conducted in the absence of any commercial or financial relationships that could be construed as a potential conflict of interest.

## Publisher’s Note

All claims expressed in this article are solely those of the authors and do not necessarily represent those of their affiliated organizations, or those of the publisher, the editors and the reviewers. Any product that may be evaluated in this article, or claim that may be made by its manufacturer, is not guaranteed or endorsed by the publisher.

## Footnotes

1
http://www.genome.ad.jp/kegg

2
https://www.wikipathways.org/
