# Supplementary material for: DNA Methylation Patterns in CD8+ T Cells Discern Psoriasis From Psoriatic Arthritis and Correlate With Cutaneous Disease Activity
Source: Front Cell Dev Biol. 2021 Oct 21;9:746145. doi: 10.3389/fcell.2021.746145 (PMC8567019; doi:10.3389/fcell.2021.746145)
Supplement: Supplementary file 4 [file Data_Sheet_1.docx]

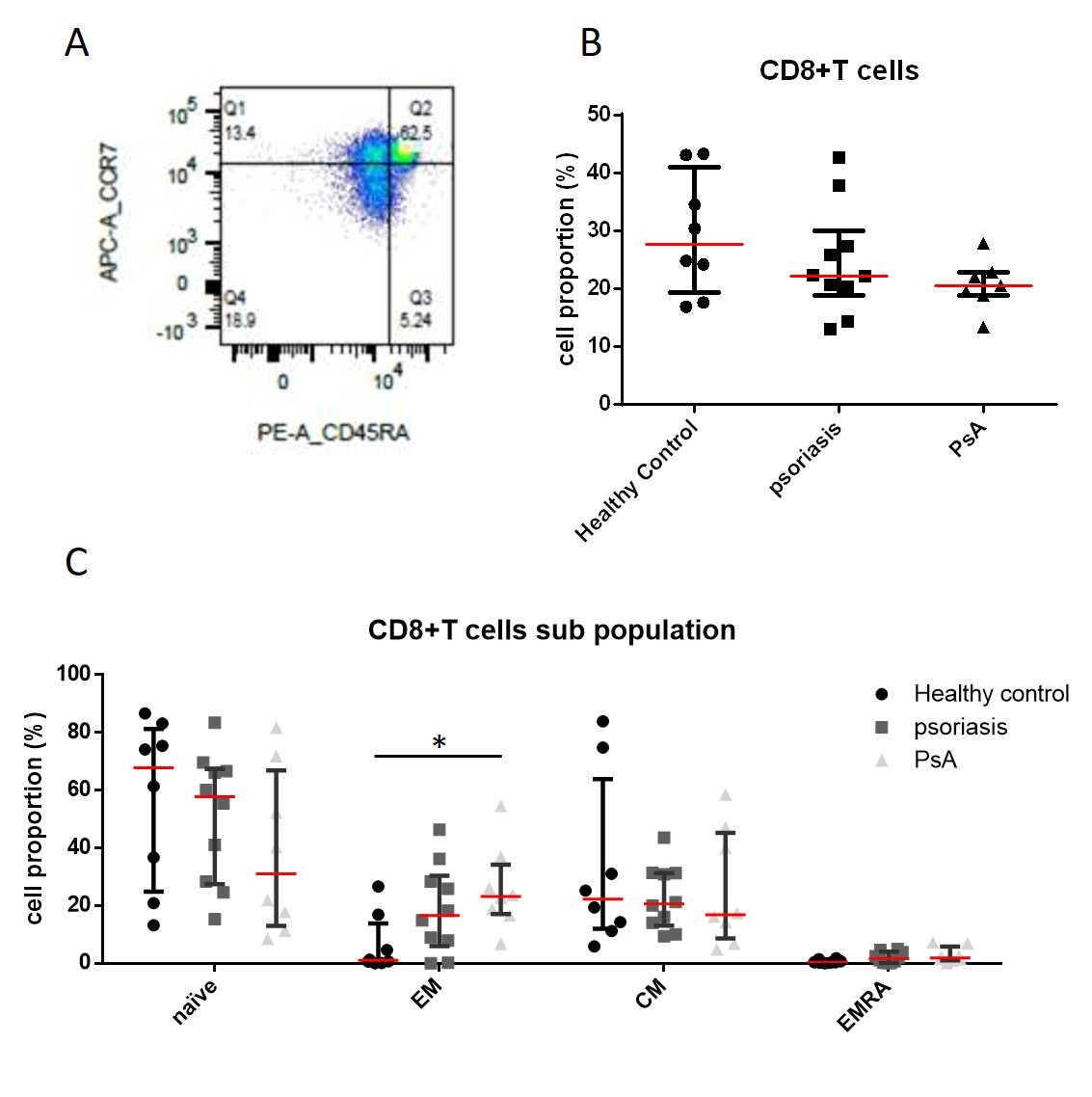


**Supplementary Figure 1: Peripheral CD8^+^ T cells phenotypes in psoriasis patients, PsA and healthy controls**. A) gating strategy apply for CD8+ T cells selection. B) CD8 +T cells global proportion between Healthy control, skin psoriasis and PsA patients. C) CD8+T cells sub population distribution in Healthy control, skin psoriasis and PsA patients. Median (red) and interquartile are represented in scatter plots. * p≥0.05, Tukey's multiple comparisons test. EM: Effector Memory, CM: Circulating central memory and EMRA: effector memory T cells that re‐express CD45RA.


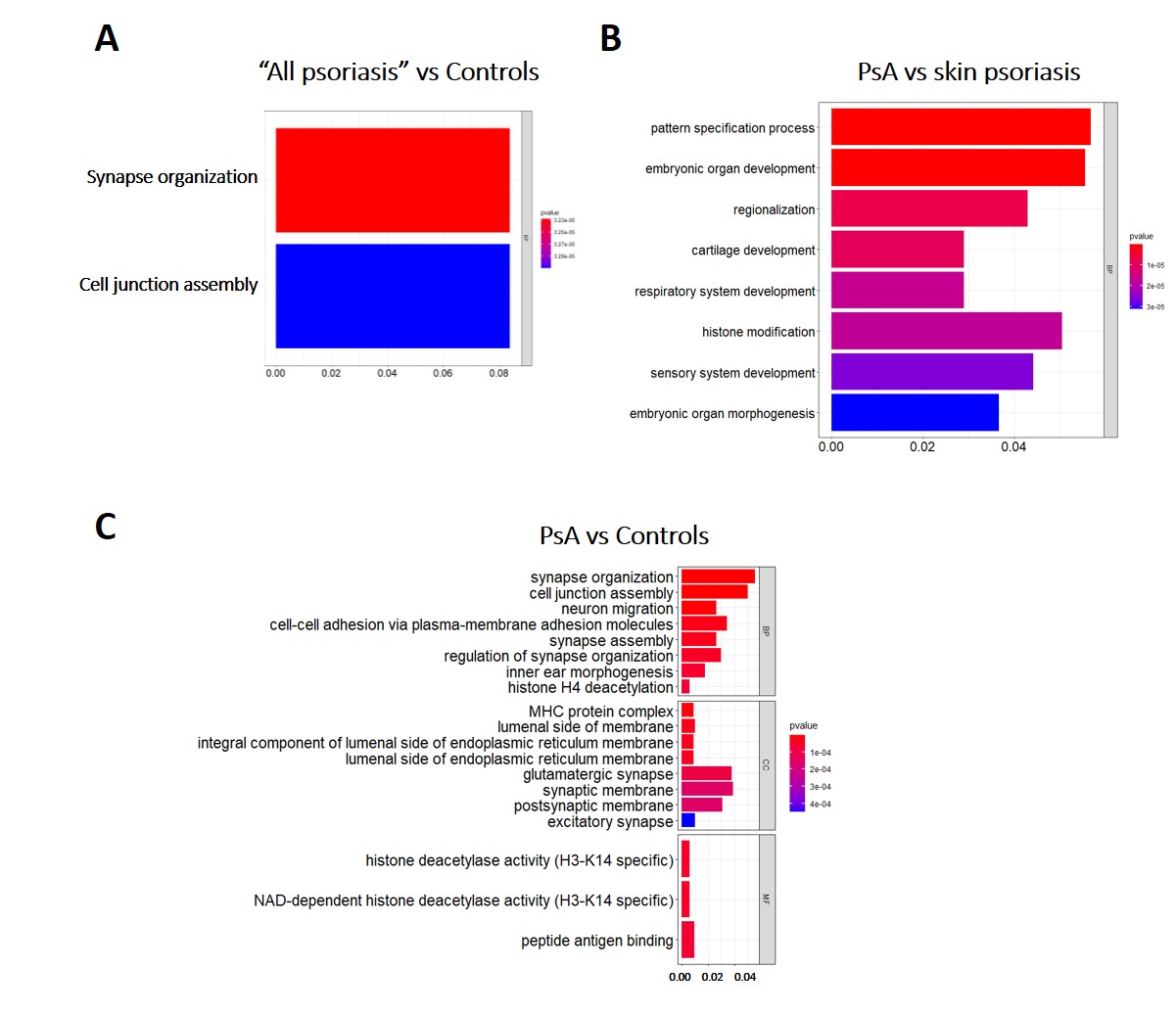


**Supplementary Figure 2: GO analyses of genes presenting DMP in both promoters and gene bodies.** Only significantly enriched terms for Biological Process (BP), Cellular Component (CC) and Molecular Function (MF) are represented (P < 0.05), in “all psoriasis” versus Controls (A), PsA versus skin psoriasis (B) and PsA versus Controls (C).

**Supplementary Table 3: Differentially Methylated Regions (DMRs in CD8+T cells from psoriasis, psoriatic arthritis (PsA) and healthy control (Control) patients with a minimum number of 5 CpG per region.**

| **All vs Control** | | | | | | | | | | | | | |
| --- | --- | --- | --- | --- | --- | --- | --- | --- | --- | --- | --- | --- | --- |
| DMR | seqnames | start | end | width | strand | no.cpgs | min_smoothed_fdr | Stouffer | HMFDR | Fisher | maxdiff | meandiff | overlapping.genes |
| 1 | chr9 | 124988720 | 124991656 | 2937 | * | 27 | 3.19E-65 | 1.40E-17 | 0.00900403 | 5.47E-17 | -0.314329782 | -0.110474059 | LHX6 |
| 2 | chr5 | 135415129 | 135416613 | 1485 | * | 17 | 4.58E-24 | 3.01E-09 | 0.069184444 | 1.43E-06 | 0.190753037 | 0.119934792 | SNORA27, SNORA68, SNORA57, 7SK, VTRNA2-1, SNORD45, SNORD95 |
| 3 | chr19 | 21264371 | 21265421 | 1051 | * | 8 | 2.06E-18 | 3.17E-07 | 0.036300526 | 1.06E-05 | -0.295609599 | -0.170086988 | ZNF714 |
| 4 | chr14 | 69095057 | 69095679 | 623 | * | 7 | 2.42E-14 | 7.32E-06 | 0.042035372 | 0.00011837 | -0.148241703 | -0.10235689 | RAD51B, SNORA46, SNORA79, CTD-2325P2.4 |
| 5 | chr17 | 4804104 | 4804838 | 735 | * | 5 | 6.77E-12 | 0.000232158 | 0.042166378 | 0.00120343 | 0.187134406 | 0.100291936 | C17orf107, CHRNE |
| 6 | chr2 | 128453108 | 128453484 | 377 | * | 5 | 3.44E-10 | 0.000199132 | 0.050144344 | 0.001286272 | -0.175307278 | -0.118134454 | SNORA12, SNORA74, snosnR60_Z15, snR65, 5S_rRNA, SNORA4, SNORD11, SNORD51, SNORA41, SCARNA6, SNORD39, SNORA75, ACA59, SNORA48, SNORD56, SNORA43, SNORA1, Vault |
| 7 | chr15 | 66947066 | 66947617 | 552 | * | 7 | 8.30E-10 | 0.00068763 | 0.068499547 | 0.003821278 | -0.191382731 | -0.135266892 | RP11-321F6.1 |
| 8 | chr1 | 248100183 | 248100614 | 432 | * | 8 | 1.51E-10 | 0.005633197 | 0.101770338 | 0.015694962 | -0.198321203 | -0.138599746 | Y_RNA, OR2L13, RP11-438H8.8 |
| 9 | chr6 | 29648736 | 29649092 | 357 | * | 6 | 3.51E-10 | 0.025682552 | 0.138103632 | 0.059361824 | -0.170631796 | -0.111211681 | ZFP57, SNORA20 |
| **Psoriasis vs Control** | | | | | | | | | | | | | |
|  | seqnames | start | end | width | strand | no.cpgs | min_smoothed_fdr | Stouffer | HMFDR | Fisher | maxdiff | meandiff | overlapping.genes |
| 1 | chr19 | 21264131 | 21265421 | 1291 | * | 10 | 1.11E-115 | 8.90E-22 | 3.62E-05 | 3.03E-24 | 0.474643783 | 0.224508323 | ZNF714 |
| 2 | chr9 | 124988720 | 124991656 | 2937 | * | 27 | 9.28E-36 | 3.52E-14 | 0.015692598 | 1.58E-13 | 0.313187205 | 0.105462159 | LHX6 |
| 3 | chr2 | 128453108 | 128453870 | 763 | * | 6 | 5.78E-45 | 1.39E-12 | 0.00028818 | 1.78E-12 | 0.276861865 | 0.168045291 | SNORA12, SNORA74, snosnR60_Z15, snR65, 5S_rRNA, SNORA4, SNORD11, SNORD51, SNORA41, SCARNA6, SNORD39, SNORA75, ACA59, SNORA48, SNORD56, SNORA43, SNORA1, Vault |
| 4 | chr1 | 24866581 | 24868592 | 2012 | * | 5 | 1.98E-29 | 1.67E-11 | 6.94E-06 | 7.59E-12 | 0.224480342 | 0.136687145 | snoU13, Y_RNA, SCARNA16, SCARNA21, U1, RCAN3, SCARNA17, SCARNA18, SNORD112, SNORA62, SNORA63, SNORD46, SNORA2, SNORD81, U3, SNORA51, SCARNA20, SNORA67, U6, SNORA70, SNORA77, SNORA26, U8, SCARNA11, RP4-594I10.3, SNORA31, SNORA42, SNORA40, ACA64, snoU109, SNORD60 |
| 5 | chr12 | 297356 | 298484 | 1129 | * | 6 | 1.16E-31 | 1.57E-10 | 0.000504934 | 8.93E-10 | -0.277844129 | -0.118915936 | NA |
| 6 | chr20 | 30134929 | 30136329 | 1401 | * | 9 | 1.84E-23 | 1.32E-11 | 0.009802438 | 1.49E-09 | -0.164983303 | -0.116640472 | HM13, RP3-324O17.4 |
| 7 | chr7 | 127910494 | 127912372 | 1879 | * | 9 | 6.80E-26 | 4.46E-10 | 0.005619162 | 1.81E-09 | -0.199281977 | -0.10136855 | NA |
| 8 | chr19 | 39997622 | 39998930 | 1309 | * | 6 | 1.98E-29 | 4.85E-09 | 0.000341408 | 9.63E-09 | -0.17135053 | -0.103646815 | DLL3 |
| 9 | chr5 | 1867978 | 1868738 | 761 | * | 8 | 4.31E-25 | 9.79E-09 | 0.005874037 | 1.15E-08 | -0.197128442 | -0.126880345 | NA |
| 10 | chr17 | 154410 | 155045 | 636 | * | 5 | 3.65E-25 | 2.98E-08 | 0.001198165 | 4.77E-08 | 0.236251901 | 0.168273751 | RPH3AL |
| 11 | chr5 | 135415129 | 135416613 | 1485 | * | 17 | 1.87E-22 | 2.27E-10 | 0.049796352 | 1.25E-07 | -0.241723111 | -0.14188436 | SNORA27, SNORA68, SNORA57, 7SK, VTRNA2-1, SNORD45, SNORD95 |
| 12 | chr4 | 2366555 | 2367137 | 583 | * | 7 | 2.96E-22 | 9.54E-06 | 0.002466559 | 2.97E-06 | 0.241590373 | 0.108610684 | ZFYVE28 |
| 13 | chr15 | 23377763 | 23378814 | 1052 | * | 5 | 1.08E-18 | 3.46E-06 | 0.002123209 | 4.91E-06 | 0.18883196 | 0.106369478 | HERC2P2 |
| 14 | chr9 | 130954250 | 130955436 | 1187 | * | 6 | 1.75E-13 | 2.51E-06 | 0.013417464 | 1.90E-05 | 0.181514828 | 0.108086882 | CIZ1 |
| 15 | chr19 | 54566838 | 54567279 | 442 | * | 6 | 2.58E-13 | 1.65E-06 | 0.026491029 | 2.54E-05 | -0.167089648 | -0.109426026 | VSTM1 |
| 16 | chr17 | 9018806 | 9019336 | 531 | * | 5 | 4.41E-12 | 1.92E-05 | 0.014985201 | 9.82E-05 | -0.189206767 | -0.109253034 | SNORA69, NTN1 |
| 17 | chr2 | 162100495 | 162101506 | 1012 | * | 6 | 2.99E-11 | 6.33E-05 | 0.015920935 | 0.000156291 | 0.174057798 | 0.107584502 | AC009299.3, 5S_rRNA, SNORA4, SNORD11, SNORD51, SNORA41, SCARNA6, SNORD39, SNORA75, ACA59, SNORA48, AC009299.2, SNORA43, SNORA1, Vault |
| 18 | chr14 | 69095057 | 69095679 | 623 | * | 7 | 3.28E-09 | 8.51E-05 | 0.052418976 | 0.000880961 | 0.150299375 | 0.10384017 | RAD51B, SNORA46, SNORA79, CTD-2325P2.4 |
| 19 | chr12 | 4918848 | 4919591 | 744 | * | 6 | 1.87E-10 | 0.000296047 | 0.030692499 | 0.001295773 | -0.136510568 | -0.112587685 | GALNT8, KCNA6 |
| 20 | chr5 | 1594330 | 1595048 | 719 | * | 10 | 3.73E-10 | 0.001935809 | 0.067750892 | 0.002100118 | 0.2360367 | 0.129985119 | CTD-2012J19.3, SDHAP3 |
| **PsA vs Control** | | | | | | | | | | | | | |
|  | seqnames | start | end | width | strand | no.cpgs | min_smoothed_fdr | Stouffer | HMFDR | Fisher | maxdiff | meandiff | overlapping.genes |
| 1 | chr19 | 21264131 | 21265421 | 1291 | * | 10 | 2.73E-61 | 4.32E-14 | 0.000908785 | 6.12E-15 | -0.324855163 | -0.149460864 | ZNF714 |
| 2 | chr19 | 55475879 | 55478588 | 2710 | * | 14 | 7.42E-34 | 1.68E-14 | 0.007328943 | 1.82E-12 | 0.196960478 | 0.126500917 | NLRP2, NLRP7 |
| 3 | chr19 | 39997622 | 39998930 | 1309 | * | 6 | 6.18E-37 | 5.12E-09 | 0.000195934 | 4.91E-09 | 0.169458044 | 0.107771573 | DLL3 |
| 4 | chr2 | 128453108 | 128453870 | 763 | * | 6 | 8.09E-23 | 1.23E-07 | 0.006121495 | 3.68E-07 | -0.221297702 | -0.119939949 | SNORA12, SNORA74, snosnR60_Z15, snR65, 5S_rRNA, SNORA4, SNORD11, SNORD51, SNORA41, SCARNA6, SNORD39, SNORA75, ACA59, SNORA48, SNORD56, SNORA43, SNORA1, Vault |
| 5 | chr8 | 215923 | 216788 | 866 | * | 5 | 8.20E-18 | 7.95E-06 | 0.007185 | 9.20E-06 | -0.200176515 | -0.136916611 | NA |
| 6 | chr17 | 154410 | 155045 | 636 | * | 5 | 6.95E-19 | 0.000122284 | 0.00420583 | 1.14E-05 | -0.194048726 | -0.139846461 | RPH3AL |
| 7 | chr2 | 162099970 | 162101506 | 1537 | * | 7 | 4.84E-13 | 2.98E-06 | 0.017268746 | 3.59E-05 | -0.190198544 | -0.128299048 | AC009299.3, 5S_rRNA, SNORA4, SNORD11, SNORD51, SNORA41, SCARNA6, SNORD39, SNORA75, ACA59, SNORA48, AC009299.2, SNORA43, SNORA1, Vault |
| 8 | chr5 | 171056823 | 171057575 | 753 | * | 7 | 5.52E-14 | 1.58E-05 | 0.024326641 | 9.42E-05 | -0.204622462 | -0.1026035 | SNORA57, SNORD95 |
| 9 | chr12 | 96617777 | 96618698 | 922 | * | 5 | 1.73E-10 | 1.71E-05 | 0.026237174 | 0.000139124 | -0.231748649 | -0.137117882 | ELK3, SNORA9, snoMe28S-Am2634 |
| 10 | chr7 | 39170497 | 39171113 | 617 | * | 6 | 3.23E-10 | 3.96E-05 | 0.049261673 | 0.000421293 | -0.183213524 | -0.11764737 | POU6F2, SNORA15 |
| 11 | chr16 | 55866757 | 55867301 | 545 | * | 6 | 2.67E-10 | 0.000179932 | 0.040387351 | 0.00080694 | -0.159159349 | -0.104716921 | SNORD111, CES1, SNORD33 |
| 12 | chr16 | 83170651 | 83171314 | 664 | * | 5 | 1.16E-09 | 0.000331285 | 0.042774797 | 0.001328003 | 0.211968025 | 0.10797138 | CDH13 |
| 13 | chr12 | 31271783 | 31272119 | 337 | * | 5 | 3.70E-09 | 0.002780286 | 0.033934808 | 0.003770795 | -0.170644549 | -0.102298357 | RP11-551L14.1, snoMe28S-Am2634 |
| 14 | chr17 | 72462164 | 72463080 | 917 | * | 12 | 1.00E-08 | 0.000620727 | 0.1484402 | 0.008962283 | -0.193152212 | -0.113900241 | SNORA69, CD300A |
| 15 | chr1 | 205818956 | 205819492 | 537 | * | 10 | 6.23E-07 | 0.007911488 | 0.202862959 | 0.059707934 | -0.214668594 | -0.160333573 | snoU13, Y_RNA, SNORD112, U3, SNORA51, SNORA25, SNORA70, SNORA26, SNORA72, U8, PM20D1, SNORD60, SNORD116 |
| **PsA vs Psoriasis** | | | | | | | | | | | | | |
|  | seqnames | start | end | width | strand | no.cpgs | min_smoothed_fdr | Stouffer | HMFDR | Fisher | maxdiff | meandiff | overlapping.genes |
| 1 | chr19 | 21264131 | 21265421 | 1291 | * | 10 | 1.11E-115 | 8.90E-22 | 3.62E-05 | 3.03E-24 | -0.474643783 | -0.224508323 | ZNF714 |
| 2 | chr9 | 124988720 | 124991656 | 2937 | * | 27 | 9.28E-36 | 3.52E-14 | 0.015692598 | 1.58E-13 | -0.313187205 | -0.105462159 | LHX6 |
| 3 | chr2 | 128453108 | 128453870 | 763 | * | 6 | 5.78E-45 | 1.39E-12 | 0.00028818 | 1.78E-12 | -0.276861865 | -0.168045291 | SNORA12, SNORA74, snosnR60_Z15, snR65, 5S_rRNA, SNORA4, SNORD11, SNORD51, SNORA41, SCARNA6, SNORD39, SNORA75, ACA59, SNORA48, SNORD56, SNORA43, SNORA1, Vault |
| 4 | chr1 | 24866581 | 24868592 | 2012 | * | 5 | 1.98E-29 | 1.67E-11 | 6.94E-06 | 7.59E-12 | -0.224480342 | -0.136687145 | snoU13, Y_RNA, SCARNA16, SCARNA21, U1, RCAN3, SCARNA17, SCARNA18, SNORD112, SNORA62, SNORA63, SNORD46, SNORA2, SNORD81, U3, SNORA51, SCARNA20, SNORA67, U6, SNORA70, SNORA77, SNORA26, U8, SCARNA11, RP4-594I10.3, SNORA31, SNORA42, SNORA40, ACA64, snoU109, SNORD60 |
| 5 | chr12 | 297356 | 298484 | 1129 | * | 6 | 1.16E-31 | 1.57E-10 | 0.000504934 | 8.93E-10 | 0.277844129 | 0.118915936 | NA |
| 6 | chr20 | 30134929 | 30136329 | 1401 | * | 9 | 1.84E-23 | 1.32E-11 | 0.009802438 | 1.49E-09 | 0.164983303 | 0.116640472 | HM13, RP3-324O17.4 |
| 7 | chr7 | 127910494 | 127912372 | 1879 | * | 9 | 6.80E-26 | 4.46E-10 | 0.005619162 | 1.81E-09 | 0.199281977 | 0.10136855 | NA |
| 8 | chr19 | 39997622 | 39998930 | 1309 | * | 6 | 1.98E-29 | 4.85E-09 | 0.000341408 | 9.63E-09 | 0.17135053 | 0.103646815 | DLL3 |
| 9 | chr5 | 1867978 | 1868738 | 761 | * | 8 | 4.31E-25 | 9.79E-09 | 0.005874037 | 1.15E-08 | 0.197128442 | 0.126880345 | NA |
| 10 | chr17 | 154410 | 155045 | 636 | * | 5 | 3.65E-25 | 2.98E-08 | 0.001198165 | 4.77E-08 | -0.236251901 | -0.168273751 | RPH3AL |
| 11 | chr5 | 135415129 | 135416613 | 1485 | * | 17 | 1.87E-22 | 2.27E-10 | 0.049796352 | 1.25E-07 | 0.241723111 | 0.14188436 | SNORA27, SNORA68, SNORA57, 7SK, VTRNA2-1, SNORD45, SNORD95 |
| 12 | chr4 | 2366555 | 2367137 | 583 | * | 7 | 2.96E-22 | 9.54E-06 | 0.002466559 | 2.97E-06 | -0.241590373 | -0.108610684 | ZFYVE28 |
| 13 | chr15 | 23377763 | 23378814 | 1052 | * | 5 | 1.08E-18 | 3.46E-06 | 0.002123209 | 4.91E-06 | -0.18883196 | -0.106369478 | HERC2P2 |
| 14 | chr9 | 130954250 | 130955436 | 1187 | * | 6 | 1.75E-13 | 2.51E-06 | 0.013417464 | 1.90E-05 | -0.181514828 | -0.108086882 | CIZ1 |
| 15 | chr19 | 54566838 | 54567279 | 442 | * | 6 | 2.58E-13 | 1.65E-06 | 0.026491029 | 2.54E-05 | 0.167089648 | 0.109426026 | VSTM1 |
| 16 | chr17 | 9018806 | 9019336 | 531 | * | 5 | 4.41E-12 | 1.92E-05 | 0.014985201 | 9.82E-05 | 0.189206767 | 0.109253034 | SNORA69, NTN1 |
| 17 | chr2 | 162100495 | 162101506 | 1012 | * | 6 | 2.99E-11 | 6.33E-05 | 0.015920935 | 0.000156291 | -0.174057798 | -0.107584502 | AC009299.3, 5S_rRNA, SNORA4, SNORD11, SNORD51, SNORA41, SCARNA6, SNORD39, SNORA75, ACA59, SNORA48, AC009299.2, SNORA43, SNORA1, Vault |
| 18 | chr14 | 69095057 | 69095679 | 623 | * | 7 | 3.28E-09 | 8.51E-05 | 0.052418976 | 0.000880961 | -0.150299375 | -0.10384017 | RAD51B, SNORA46, SNORA79, CTD-2325P2.4 |
| 19 | chr12 | 4918848 | 4919591 | 744 | * | 6 | 1.87E-10 | 0.000296047 | 0.030692499 | 0.001295773 | 0.136510568 | 0.112587685 | GALNT8, KCNA6 |
| 20 | chr5 | 1594330 | 1595048 | 719 | * | 10 | 3.73E-10 | 0.001935809 | 0.067750892 | 0.002100118 | -0.2360367 | -0.129985119 | CTD-2012J19.3, SDHAP3 |

**Supplementary Table 4: Differentially Methylated Regions (DMRs in CD8+T cells from psoriasis, psoriatic arthritis (PsA) and healthy control (Control) patients with a minimum number of 10 CpG per region.**

| **All vs Control** | | | | | | | | | | | | | |
| --- | --- | --- | --- | --- | --- | --- | --- | --- | --- | --- | --- | --- | --- |
|  | seqnames | start | end | width | strand | no.cpgs | min_smoothed_fdr | Stouffer | HMFDR | Fisher | maxdiff | meandiff | overlapping.genes |
| 1 | chr9 | 124988720 | 124991656 | 2937 | * | 27 | 3.19E-65 | 1.40E-17 | 0.00900403 | 5.47E-17 | -0.31433 | -0.110474059 | LHX6 |
| 2 | chr5 | 135415129 | 135416613 | 1485 | * | 17 | 4.58E-24 | 3.01E-09 | 0.069184444 | 1.43E-06 | 0.190753 | 0.119934792 | SNORA27, SNORA68, SNORA57, 7SK, VTRNA2-1, SNORD45, SNORD95 |
| **Psoriasis vs Control** | | | | | | | | | | | | | |
|  | seqnames | start | end | width | strand | no.cpgs | min_smoothed_fdr | Stouffer | HMFDR | Fisher | maxdiff | meandiff | overlapping.genes |
| 1 | chr19 | 21264131 | 21265421 | 1291 | * | 10 | 1.11E-115 | 8.90E-22 | 3.62E-05 | 3.03E-24 | 0.474644 | 0.224508323 | ZNF714 |
| 2 | chr9 | 124988720 | 124991656 | 2937 | * | 27 | 9.28E-36 | 3.52E-14 | 0.015692598 | 1.58E-13 | 0.313187 | 0.105462159 | LHX6 |
| 3 | chr5 | 135415129 | 135416613 | 1485 | * | 17 | 1.87E-22 | 2.27E-10 | 0.049796352 | 1.25E-07 | -0.24172 | -0.14188436 | SNORA27, SNORA68, SNORA57, 7SK, VTRNA2-1, SNORD45, SNORD95 |
| 4 | chr5 | 1594330 | 1595048 | 719 | * | 10 | 3.73E-10 | 0.001935809 | 0.067750892 | 0.0021 | 0.236037 | 0.129985119 | CTD-2012J19.3, SDHAP3 |
| **PsA vs Control** | | | | | | | | | | | | | |
|  | seqnames | start | end | width | strand | no.cpgs | min_smoothed_fdr | Stouffer | HMFDR | Fisher | maxdiff | meandiff | overlapping.genes |
| 1 | chr19 | 21264131 | 21265421 | 1291 | * | 10 | 2.73E-61 | 4.32E-14 | 0.000908785 | 6.12E-15 | -0.32486 | -0.149460864 | ZNF714 |
| 2 | chr19 | 55475879 | 55478588 | 2710 | * | 14 | 7.42E-34 | 1.68E-14 | 0.007328943 | 1.82E-12 | 0.19696 | 0.126500917 | NLRP2, NLRP7 |
| 3 | chr17 | 72462164 | 72463080 | 917 | * | 12 | 1.00E-08 | 0.000620727 | 0.1484402 | 0.008962 | -0.19315 | -0.113900241 | SNORA69, CD300A |
| 4 | chr1 | 205818956 | 205819492 | 537 | * | 10 | 6.23E-07 | 0.007911488 | 0.202862959 | 0.059708 | -0.21467 | -0.160333573 | snoU13, Y_RNA, SNORD112, U3, SNORA51, SNORA25, SNORA70, SNORA26, SNORA72, U8, PM20D1, SNORD60, SNORD116 |
| **PsA vs Psoriasis** | | | | | | | | | | | | | |
|  | seqnames | start | end | width | strand | no.cpgs | min_smoothed_fdr | Stouffer | HMFDR | Fisher | maxdiff | meandiff | overlapping.genes |
| 1 | chr19 | 21264131 | 21265421 | 1291 | * | 10 | 1.11E-115 | 8.90E-22 | 3.62E-05 | 3.03E-24 | -0.47464 | -0.224508323 | ZNF714 |
| 2 | chr9 | 124988720 | 124991656 | 2937 | * | 27 | 9.28E-36 | 3.52E-14 | 0.015692598 | 1.58E-13 | -0.31319 | -0.105462159 | LHX6 |
| 3 | chr5 | 135415129 | 135416613 | 1485 | * | 17 | 1.87E-22 | 2.27E-10 | 0.049796352 | 1.25E-07 | 0.241723 | 0.14188436 | SNORA27, SNORA68, SNORA57, 7SK, VTRNA2-1, SNORD45, SNORD95 |
| 4 | chr5 | 1594330 | 1595048 | 719 | * | 10 | 3.73E-10 | 0.001935809 | 0.067750892 | 0.0021 | -0.23604 | -0.129985119 | CTD-2012J19.3, SDHAP3 |
|  |  |  |  |  |  |  |  |  |  |  |  |  |  |

**Supplementary Table 5: Differentially Methylated Regions (DMRs in CD8+T cells from psoriasis, psoriatic arthritis (PsA) and healthy control (Control) patients with a minimum number of 20 CpG per region.**

| **All vs control** | | | | | | | | | | | | | |
| --- | --- | --- | --- | --- | --- | --- | --- | --- | --- | --- | --- | --- | --- |
|  | seqnames | start | end | width | strand | no.cpgs | min_smoothed_fdr | Stouffer | HMFDR | Fisher | maxdiff | meandiff | overlapping.genes |
| 1 | chr9 | 1.25E+08 | 1.25E+08 | 2937 | * | 27 | 3.19E-65 | 1.40E-17 | 0.009004 | 5.47E-17 | -0.31433 | -0.11047 | LHX6 |
| **Psoriasis vs Control** | | | | | | | | | | | | | |
|  | seqnames | start | end | width | strand | no.cpgs | min_smoothed_fdr | Stouffer | HMFDR | Fisher | maxdiff | meandiff | overlapping.genes |
| 1 | chr9 | 1.25E+08 | 1.25E+08 | 2937 | * | 27 | 9.28E-36 | 3.52E-14 | 0.015693 | 1.58E-13 | 0.313187 | 0.105462 | LHX6 |
| **PsA vs Psoriasis** | | | | | | | | | | | | | |
|  | seqnames | start | end | width | strand | no.cpgs | min_smoothed_fdr | Stouffer | HMFDR | Fisher | maxdiff | meandiff | overlapping.genes |
| 1 | chr9 | 1.25E+08 | 1.25E+08 | 2937 | * | 27 | 9.28E-36 | 3.52E-14 | 0.015693 | 1.58E-13 | -0.31319 | -0.10546 | LHX6 |


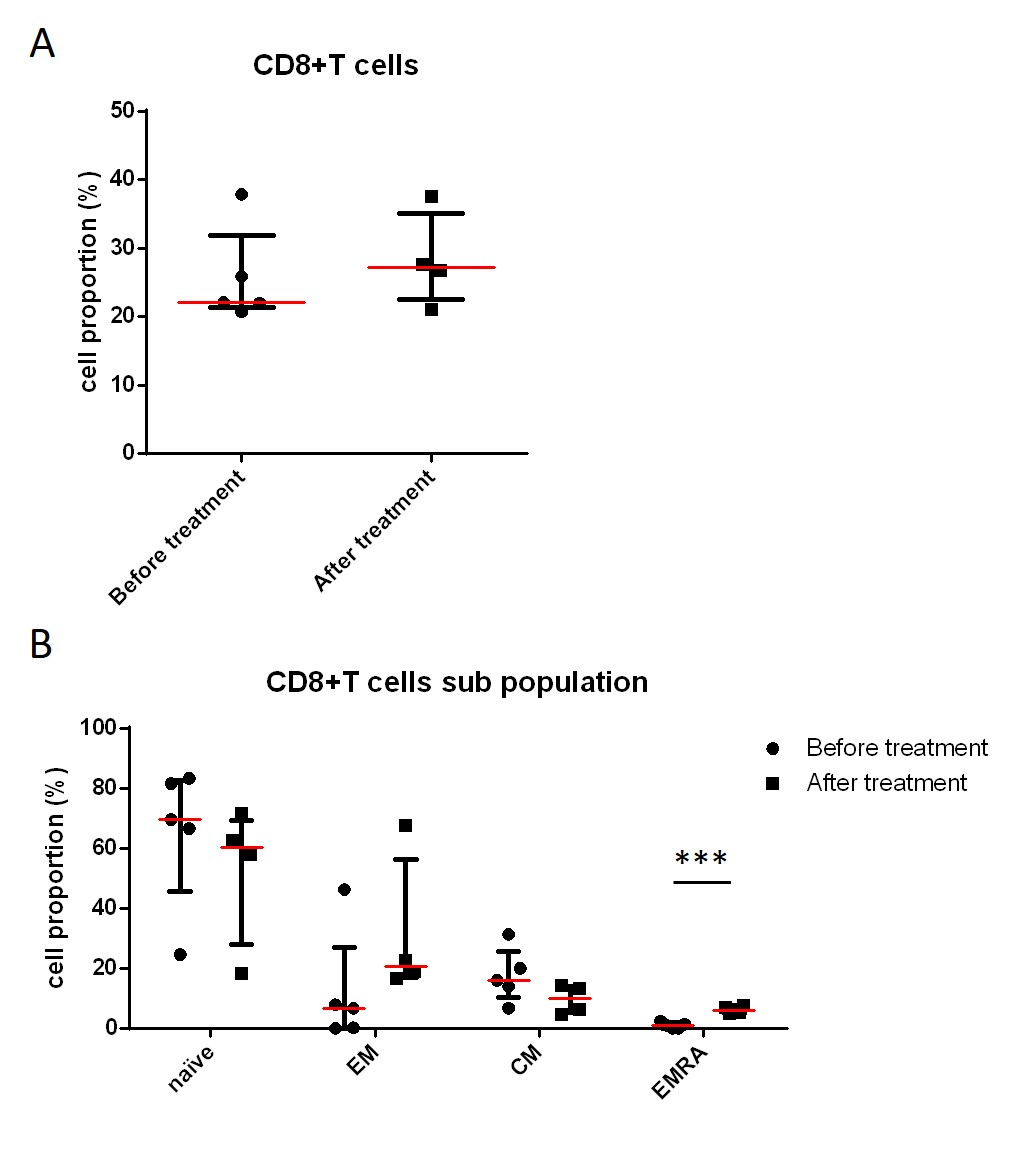


**Supplementary Figure 3: Peripheral CD8^+^ T cells phenotypes in patients before and after treatment**.

A) CD8 +T cells global proportion between Healthy control, skin psoriasis and PsA patients. B) CD8+T cells sub population distribution in Healthy control, skin psoriasis and PsA patients. Median (red) and interquartile are represented in scatter plots.

*** p=0.003, Tukey's multiple comparisons test. EM: Effector Memory, CM: Circulating central memory and EMRA: effector memory T cells that re‐express CD45RA.


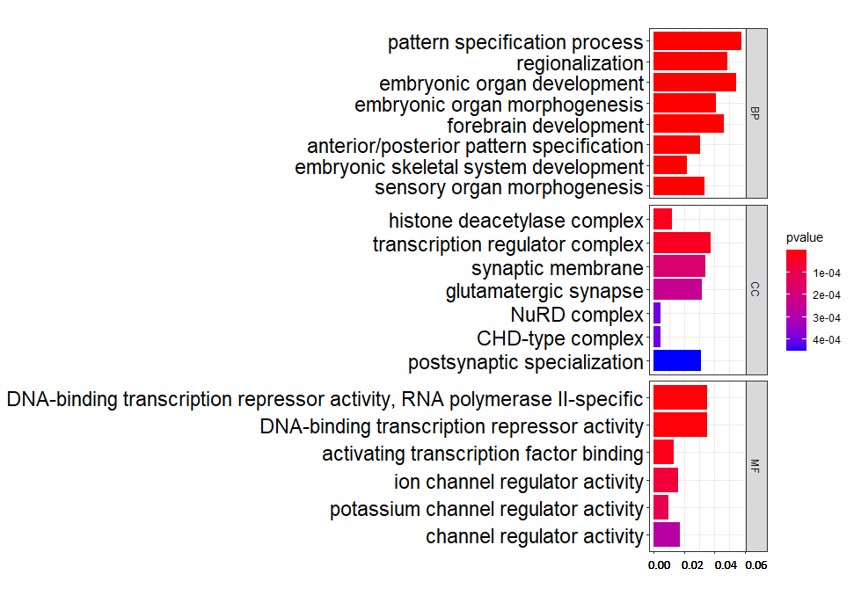


**Supplementary Figure 4: GO analyses of genes presenting DMP in both promoters and gene bodies in response to treatment.** Only significantly enriched terms for Biological Process (BP), Cellular Component (CC) and Molecular Function (MF) are represented (P < 0.05).

**Supplementary Table 6:** **Significant GO categories selected from the analysis with clusterProfiler of the identified genes carrier at least 1 DMP in their promoter (TSS1500, TSS200, 5’UTR) in patient in response to treatment.**

|  | ONTOLOGY | ID | Description | GeneRatio | BgRatio | pvalue | p.adjust | qvalue | geneID | Count |
| --- | --- | --- | --- | --- | --- | --- | --- | --- | --- | --- |
| GO:0048562 | BP | GO:0048562 | embryonic organ morphogenesis | 42/998 | 302/18866 | 9.57E-07 | 0.00499 | 0.00471 | 55084/2303/23314/6664/2778/2290/2295/5013/388389/7545/2253/4487/4088/7481/10716/220/388591/123872/652/3227/4087/6670/6909/286262/10413/1739/2260/344022/11078/79977/5079/161497/3221/5080/6913/4323/64321/2535/3213/51384/5915/157506 | 42 |
| GO:0048568 | BP | GO:0048568 | embryonic organ development | 51/998 | 451/18866 | 2.61E-05 | 595638 | 561703 | 55084/2303/6886/23314/6664/2778/7262/2290/2295/5013/388389/7545/2253/4487/4088/7481/9394/10716/220/388591/123872/652/2668/3227/203447/4087/6670/6909/2186/196528/286262/10413/1739/7005/2260/3481/344022/11078/79977/5079/161497/3221/5080/6913/4323/64321/2535/3213/51384/5915/157506 | 51 |
| GO:0007389 | BP | GO:0007389 | pattern specification process | 51/998 | 455/18866 | 3.43E-05 | 595638 | 561703 | 2303/3234/23314/140628/2290/8313/5013/6790/55502/388389/7545/2253/10468/4487/4825/5015/4088/30062/7481/4300/10716/388591/123872/652/8553/2668/4488/3227/5649/80173/4087/6909/2186/22955/3670/2018/4821/2260/3238/344022/8022/3222/3221/4681/80319/5080/63950/7516/64321/54880/3213 | 51 |
| GO:0003002 | BP | GO:0003002 | regionalization | 41/998 | 355/18866 | 0.000227 | 2555463 | 2409875 | 2303/3234/140628/2290/8313/5013/6790/55502/2253/4487/4825/5015/4088/7481/4300/10716/123872/652/8553/2668/4488/3227/5649/4087/2186/22955/3670/2018/4821/2260/344022/8022/3222/3221/4681/80319/5080/63950/7516/64321/3213 | 41 |
| GO:0003007 | BP | GO:0003007 | heart morphogenesis | 33/998 | 258/18866 | 0.000245 | 2555463 | 2409875 | 2303/6664/140628/57178/7273/8313/388389/2253/4487/7134/4088/9353/7481/8751/10716/388591/123872/652/4488/6909/196528/3670/10413/4092/344022/79977/4734/4886/4889/64321/2535/51384/5915 | 33 |
| GO:0023019 | BP | GO:0023019 | signal transduction involved in regulation of gene expression | 8/998 | 20/18866 | 0.000425 | 3691215 | 3480922 | 2253/4487/4088/4488/4087/5080/64321/4072 | 8 |
| GO:0061351 | BP | GO:0061351 | neural precursor cell proliferation | 23/998 | 154/18866 | 0.000633 | 4718358 | 4449547 | 85458/11023/10642/2290/2253/5803/57096/9181/5455/22911/7101/2018/64211/7005/2260/9833/6095/5108/5080/627/63950/116/4673 | 23 |
| GO:0021983 | BP | GO:0021983 | pituitary gland development | 11/998 | 42/18866 | 0.000809 | 5174851 | 4880033 | 2253/4487/219409/652/6657/3670/6658/8022/2113/5080/116 | 11 |
| GO:0010092 | BP | GO:0010092 | specification of animal organ identity | 10/998 | 36/18866 | 0.00118 | 5174851 | 4880033 | 140628/8313/2253/7481/10716/652/2668/3227/3670/2260 | 10 |
| GO:0048704 | BP | GO:0048704 | embryonic skeletal system morphogenesis | 17/998 | 97/18866 | 0.0012 | 5174851 | 4880033 | 2303/23314/6664/2778/2253/4088/652/3227/4087/1739/79977/5079/3221/6913/4323/3213/157506 | 17 |
| GO:0072132 | BP | GO:0072132 | mesenchyme morphogenesis | 12/998 | 52/18866 | 0.0013 | 5174851 | 4880033 | 2303/140628/2253/4487/4088/7481/4488/4087/6909/3670/2260/4609 | 12 |
| GO:0001657 | BP | GO:0001657 | ureteric bud development | 17/998 | 98/18866 | 0.00138 | 5174851 | 4880033 | 2303/2253/170690/4088/9353/7481/652/2668/4087/1739/2260/4092/627/4609/4072/84445/5915 | 17 |
| GO:1903845 | BP | GO:1903845 | negative regulation of cellular response to transforming growth factor beta stimulus | 16/998 | 89/18866 | 0.00154 | 5174851 | 4880033 | 23089/857/3175/7316/4088/5654/8837/56937/4087/51341/94031/203100/4092/1149/652991/114990 | 16 |
| GO:0072163 | BP | GO:0072163 | mesonephric epithelium development | 17/998 | 99/18866 | 0.00158 | 5174851 | 4880033 | 2303/2253/170690/4088/9353/7481/652/2668/4087/1739/2260/4092/627/4609/4072/84445/5915 | 17 |
| GO:0072164 | BP | GO:0072164 | mesonephric tubule development | 17/998 | 99/18866 | 0.00158 | 5174851 | 4880033 | 2303/2253/170690/4088/9353/7481/652/2668/4087/1739/2260/4092/627/4609/4072/84445/5915 | 17 |
| GO:0048706 | BP | GO:0048706 | embryonic skeletal system development | 20/998 | 130/18866 | 0.0016 | 5174851 | 4880033 | 2303/23314/6664/2778/2253/4088/7481/652/3227/4087/6670/1739/79977/5079/3222/3221/6913/4323/3213/157506 | 20 |
| GO:0007178 | BP | GO:0007178 | transmembrane receptor protein serine/threonine kinase signaling pathway | 39/998 | 359/18866 | 0.00169 | 5174851 | 4880033 | 11167/51701/6664/8425/57178/121551/23089/3622/857/3175/7316/10468/4487/4088/5654/128853/652/56937/353500/4488/4087/51341/94031/203100/6711/4092/1149/5045/652991/91851/4681/9688/4053/55512/114990/7431/2530/25970/2657 | 39 |
| GO:0090287 | BP | GO:0090287 | regulation of cellular response to growth factor stimulus | 35/998 | 310/18866 | 0.002 | 5804833 | 5474124 | 11167/4908/6664/8425/23089/3622/857/3175/7316/10468/4487/5015/4088/9353/8322/2887/5654/652/8837/56937/4488/4087/66000/51341/94031/203100/2260/4092/1149/4734/652991/91851/4681/4641/114990 | 35 |
| GO:0001823 | BP | GO:0001823 | mesonephros development | 17/998 | 103/18866 | 0.0027 | 7410586 | 6988396 | 2303/2253/170690/4088/9353/7481/652/2668/4087/1739/2260/4092/627/4609/4072/84445/5915 | 17 |
| GO:0035270 | BP | GO:0035270 | endocrine system development | 19/998 | 125/18866 | 0.00308 | 7482809 | 7056504 | 3175/2253/4487/4825/4088/7481/219409/652/6657/4087/3670/4821/6658/6009/8022/2113/5080/116/3213 | 19 |
| GO:0048701 | BP | GO:0048701 | embryonic cranial skeleton morphogenesis | 11/998 | 48/18866 | 0.00319 | 7482809 | 7056504 | 2303/2778/2253/4088/652/4087/79977/5079/6913/4323/157506 | 11 |
| GO:0048762 | BP | GO:0048762 | mesenchymal cell differentiation | 28/998 | 229/18866 | 0.0032 | 7482809 | 7056504 | 2303/6664/140628/2295/8313/2253/4487/4088/7481/8751/652/2668/152007/4488/4087/6934/3670/10509/84695/4017/2260/4092/54910/10500/114990/51384/51094/157506 | 28 |
| GO:0090092 | BP | GO:0090092 | regulation of transmembrane receptor protein serine/threonine kinase signaling pathway | 30/998 | 254/18866 | 0.0033 | 7482809 | 7056504 | 11167/6664/8425/23089/3622/857/3175/7316/10468/4487/4088/5654/652/56937/353500/4488/4087/51341/94031/203100/6711/4092/1149/652991/91851/4681/9688/114990/25970/2657 | 30 |
| GO:0050804 | BP | GO:0050804 | modulation of chemical synaptic transmission | 45/998 | 454/18866 | 0.00395 | 8257894 | 7787431 | 2899/9699/57555/10211/4908/107/152330/22941/11188/23154/8541/2668/5649/4914/154/6844/66000/2914/7101/22883/10590/1816/9627/4803/2918/2912/22997/9498/4137/627/4889/266743/9378/6863/22849/7349/5908/1269/116/57689/2917/8224/6857/9294/8526 | 45 |
| GO:0090596 | BP | GO:0090596 | sensory organ morphogenesis | 31/998 | 269/18866 | 0.00396 | 8257894 | 7787431 | 55084/6664/1735/2290/2295/23287/5013/7545/362/2253/4487/220/7068/57096/652/6670/6909/83552/286262/1826/2260/11078/161497/5080/10152/627/2535/7070/118427/51384/5915 | 31 |
| GO:0099177 | BP | GO:0099177 | regulation of trans-synaptic signaling | 45/998 | 455/18866 | 0.00417 | 8359109 | 788288 | 2899/9699/57555/10211/4908/107/152330/22941/11188/23154/8541/2668/5649/4914/154/6844/66000/2914/7101/22883/10590/1816/9627/4803/2918/2912/22997/9498/4137/627/4889/266743/9378/6863/22849/7349/5908/1269/116/57689/2917/8224/6857/9294/8526 | 45 |
| GO:0030512 | BP | GO:0030512 | negative regulation of transforming growth factor beta receptor signaling pathway | 15/998 | 87/18866 | 0.0047 | 8957735 | 8447402 | 23089/857/3175/7316/4088/5654/56937/4087/51341/94031/203100/4092/1149/652991/114990 | 15 |
| GO:1903844 | BP | GO:1903844 | regulation of cellular response to transforming growth factor beta stimulus | 19/998 | 129/18866 | 0.00481 | 8957735 | 8447402 | 6664/8425/23089/3622/857/3175/7316/4088/5654/8837/56937/4087/51341/94031/203100/4092/1149/652991/114990 | 19 |
| GO:0060485 | BP | GO:0060485 | mesenchyme development | 32/998 | 290/18866 | 0.00697 | 12052237 | 11365606 | 2303/6664/140628/2295/8313/2253/4487/4088/7481/8751/652/2668/152007/4488/4087/6934/6909/3670/10509/84695/4017/10413/2260/4092/4734/54910/10500/4609/114990/51384/51094/157506 | 32 |
| GO:0003151 | BP | GO:0003151 | outflow tract morphogenesis | 14/998 | 80/18866 | 0.00704 | 12052237 | 11365606 | 2303/6664/2253/7481/652/4488/6909/3670/4734/4886/4889/64321/2535/5915 | 14 |
| GO:0021846 | BP | GO:0021846 | cell proliferation in forebrain | 8/998 | 28/18866 | 0.00718 | 12052237 | 11365606 | 85458/10642/2253/5455/2018/64211/2260/5108 | 8 |
| GO:0045165 | BP | GO:0045165 | cell fate commitment | 31/998 | 278/18866 | 0.0074 | 12052237 | 11365606 | 2303/6886/23314/140628/2290/3175/2253/4825/7481/5468/10716/219409/652/6657/4087/6934/6909/9181/3670/7101/84695/7005/4821/2260/6095/8022/5080/6913/63950/64321/51384 | 31 |
| GO:0060541 | BP | GO:0060541 | respiratory system development | 25/998 | 204/18866 | 0.00802 | 12670825 | 11948951 | 6664/2253/94235/7481/9394/220/7068/123872/652/4087/7534/55294/6670/84695/10413/2260/79977/340419/8022/55512/54768/4323/342371/5499/157506 | 25 |
| GO:0042391 | BP | GO:0042391 | regulation of membrane potential | 43/998 | 443/18866 | 0.00941 | 14239666 | 13428414 | 2899/2273/9699/57555/27094/3753/857/22941/2567/610/8913/22953/388591/27068/63895/9780/665/5649/2562/154/66000/885/1739/10021/4092/54207/6717/22997/4734/4719/1824/9498/149111/4137/627/266743/9378/6863/1269/10052/116/9294/57030 | 43 |
| GO:0014047 | BP | GO:0014047 | glutamate secretion | 10/998 | 45/18866 | 0.00962 | 14239666 | 13428414 | 8541/6844/885/2912/7200/627/4889/2917/6857/57030 | 10 |
| GO:0007062 | BP | GO:0007062 | sister chromatid cohesion | 12/998 | 63/18866 | 0.00983 | 14239666 | 13428414 | 8313/10734/10274/55294/23137/150280/27127/2237/5885/9126/91272/55869 | 12 |
| GO:0051966 | BP | GO:0051966 | regulation of synaptic transmission, glutamatergic | 13/998 | 73/18866 | 105254 | 14835056 | 13989884 | 2899/57555/22941/5649/4914/2914/2918/2912/9378/7349/116/2917/6857 | 13 |
| GO:0030900 | BP | GO:0030900 | forebrain development | 39/998 | 391/18866 | 110992 | 15023713 | 14167793 | 85458/23314/57178/10642/2290/23287/5013/2253/4487/5015/30062/9353/10716/220/219409/27164/652/6657/5649/5455/66000/22911/3670/7101/22803/2018/64211/2260/6658/10092/5079/8022/5108/2113/5080/63950/116/5915/8925 | 39 |
| GO:0002064 | BP | GO:0002064 | epithelial cell development | 26/998 | 221/18866 | 115185 | 15023713 | 14167793 | 2303/29842/169044/3175/4825/80781/652/80173/4914/286262/10413/4821/2260/11078/6009/79977/161497/286451/5080/10152/54768/5908/7431/91663/9294/5915 | 26 |
| GO:0002066 | BP | GO:0002066 | columnar/cuboidal epithelial cell development | 12/998 | 64/18866 | 115235 | 15023713 | 14167793 | 4825/652/286262/10413/4821/2260/11078/6009/161497/286451/5080/5915 | 12 |
| GO:0001558 | BP | GO:0001558 | regulation of cell growth | 41/998 | 420/18866 | 120017 | 15097347 | 14237231 | 5931/2273/9699/9564/8425/8314/10516/4487/4825/4088/9353/7481/5468/185/8751/6049/2803/400410/23011/6446/4879/10509/22883/1826/4803/4092/136319/116224/81621/54910/4137/627/51193/10500/7349/4323/127833/64321/9166/6857/51094 | 41 |
| GO:0021536 | BP | GO:0021536 | diencephalon development | 13/998 | 74/18866 | 121589 | 15097347 | 14237231 | 5013/2253/4487/30062/219409/652/6657/3670/6658/8022/2113/5080/116 | 13 |
| GO:0017015 | BP | GO:0017015 | regulation of transforming growth factor beta receptor signaling pathway | 18/998 | 127/18866 | 125016 | 15161865 | 14298074 | 6664/8425/23089/3622/857/3175/7316/4088/5654/56937/4087/51341/94031/203100/4092/1149/652991/114990 | 18 |
| GO:0048880 | BP | GO:0048880 | sensory system development | 39/998 | 394/18866 | 130392 | 15454372 | 14573916 | 2303/6664/1735/11023/2295/23287/362/4088/30062/8322/220/5158/7068/57096/652/6657/6670/6909/3670/7101/22803/83552/1826/1739/79977/155051/91851/4867/5080/10152/627/2823/7431/7070/118427/51384/57030/5915/157506 | 39 |
| GO:0048645 | BP | GO:0048645 | animal organ formation | 12/998 | 65/18866 | 134613 | 15600152 | 14711391 | 140628/8313/2253/7481/10716/652/2668/3227/3670/2260/64321/157506 | 12 |
| GO:0035249 | BP | GO:0035249 | synaptic transmission, glutamatergic | 15/998 | 96/18866 | 149689 | 1697012 | 16003311 | 2899/57555/22941/5649/4914/2914/2918/2912/149111/9378/7349/116/2917/6857/57030 | 15 |
| GO:0001837 | BP | GO:0001837 | epithelial to mesenchymal transition | 20/998 | 152/18866 | 153581 | 17040956 | 16070111 | 2295/8313/2253/4487/4088/7481/8751/652/152007/4488/4087/6934/3670/84695/4017/2260/4092/114990/51384/51094 | 20 |
| GO:0001654 | BP | GO:0001654 | eye development | 38/998 | 384/18866 | 159102 | 17125091 | 16149452 | 2303/6664/1735/11023/2295/23287/362/4088/30062/8322/220/5158/7068/57096/652/6657/6670/6909/7101/22803/83552/1826/1739/79977/155051/91851/4867/5080/10152/627/2823/7431/7070/118427/51384/57030/5915/157506 | 38 |
| GO:0033555 | BP | GO:0033555 | multicellular organismal response to stress | 13/998 | 76/18866 | 160907 | 17125091 | 16149452 | 64856/116372/3358/22953/5649/4914/885/7101/4862/627/6863/7349/116 | 13 |
| GO:0030324 | BP | GO:0030324 | lung development | 22/998 | 177/18866 | 171096 | 17576494 | 16575138 | 6664/2253/7481/9394/7068/123872/652/4087/7534/55294/6670/84695/10413/2260/79977/340419/8022/55512/4323/342371/5499/157506 | 22 |
| GO:0001759 | BP | GO:0001759 | organ induction | 7/998 | 24/18866 | 177545 | 17576494 | 16575138 | 140628/2253/7481/652/2668/3227/2260 | 7 |
| GO:0072073 | BP | GO:0072073 | kidney epithelium development | 19/998 | 142/18866 | 178233 | 17576494 | 16575138 | 2303/2253/170690/4088/9353/7481/652/2668/4087/5455/10413/1739/2260/4092/627/4609/4072/84445/5915 | 19 |
| GO:0007179 | BP | GO:0007179 | transforming growth factor beta receptor signaling pathway | 24/998 | 202/18866 | 17863 | 17576494 | 16575138 | 51701/6664/8425/57178/23089/3622/857/3175/7316/4088/5654/128853/56937/4087/51341/94031/203100/4092/1149/5045/652991/4053/114990/2530 | 24 |
| GO:0150063 | BP | GO:0150063 | visual system development | 38/998 | 388/18866 | 196452 | 18972179 | 17891309 | 2303/6664/1735/11023/2295/23287/362/4088/30062/8322/220/5158/7068/57096/652/6657/6670/6909/7101/22803/83552/1826/1739/79977/155051/91851/4867/5080/10152/627/2823/7431/7070/118427/51384/57030/5915/157506 | 38 |
| GO:1904888 | BP | GO:1904888 | cranial skeletal system development | 12/998 | 68/18866 | 210339 | 1986337 | 18731728 | 2303/2778/2253/4088/652/4488/4087/79977/5079/6913/4323/157506 | 12 |
| GO:0021953 | BP | GO:0021953 | central nervous system neuron differentiation | 23/998 | 192/18866 | 213886 | 1986337 | 18731728 | 6886/23314/57178/2290/23287/2253/4825/9353/10716/219409/27164/3670/7101/64211/4821/136319/6095/3320/8022/5080/4137/1453/8925 | 23 |
| GO:0048592 | BP | GO:0048592 | eye morphogenesis | 20/998 | 156/18866 | 219035 | 1986337 | 18731728 | 6664/1735/2295/23287/362/220/7068/57096/652/6670/6909/83552/1826/5080/10152/627/7070/118427/51384/5915 | 20 |
| GO:0090101 | BP | GO:0090101 | negative regulation of transmembrane receptor protein serine/threonine kinase signaling pathway | 18/998 | 133/18866 | 226117 | 1986337 | 18731728 | 23089/857/3175/7316/10468/4088/5654/56937/4087/51341/94031/203100/4092/1149/652991/91851/4681/114990 | 18 |
| GO:0048339 | BP | GO:0048339 | paraxial mesoderm development | 6/998 | 18/18866 | 231245 | 1986337 | 18731728 | 2303/4088/7481/4087/10413/2260 | 6 |
| GO:0060080 | BP | GO:0060080 | inhibitory postsynaptic potential | 6/998 | 18/18866 | 231245 | 1986337 | 18731728 | 9699/57555/2562/22997/627/266743 | 6 |
| GO:0016049 | BP | GO:0016049 | cell growth | 45/998 | 490/18866 | 232342 | 1986337 | 18731728 | 5931/2273/9699/9564/8425/8314/10516/6790/4487/4825/4088/9353/7481/5468/185/8751/6049/2803/400410/23011/6446/66000/4879/10509/22883/1826/4803/4092/136319/116224/3320/81621/54910/4137/627/51193/10500/7349/4323/127833/64321/9166/6857/10611/51094 | 45 |
| GO:0030323 | BP | GO:0030323 | respiratory tube development | 22/998 | 181/18866 | 236284 | 19874543 | 18742265 | 6664/2253/7481/9394/7068/123872/652/4087/7534/55294/6670/84695/10413/2260/79977/340419/8022/55512/4323/342371/5499/157506 | 22 |
| GO:0009952 | BP | GO:0009952 | anterior/posterior pattern specification | 25/998 | 219/18866 | 247875 | 20518558 | 19349589 | 2303/3234/8313/5013/6790/55502/2253/4487/5015/4088/4300/652/8553/4488/3227/4087/2186/22955/2018/3222/3221/5080/7516/64321/3213 | 25 |
| GO:0035176 | BP | GO:0035176 | social behavior | 10/998 | 51/18866 | 288818 | 23315636 | 21987314 | 2922/22941/94235/7101/5108/266743/9378/7349/342371/9369 | 10 |
| GO:0060021 | BP | GO:0060021 | roof of mouth development | 14/998 | 91/18866 | 290607 | 23315636 | 21987314 | 23314/6664/11023/2295/4487/7481/55003/2562/4087/6909/84695/1739/2535/54880 | 14 |
| GO:0007409 | BP | GO:0007409 | axonogenesis | 44/998 | 482/18866 | 309174 | 24429451 | 23037673 | 10211/11023/7204/2290/2253/107/152330/4825/5015/5803/9353/3925/1945/1944/10716/2887/6049/2668/2803/5649/4914/23011/399/284656/885/3670/7101/10509/1796/1826/6711/4803/3320/8022/5080/54910/4137/627/10500/10752/9378/57689/9369/7070 | 44 |
| GO:0021872 | BP | GO:0021872 | forebrain generation of neurons | 11/998 | 61/18866 | 314739 | 24497982 | 23102301 | 23314/57178/2290/2253/9353/10716/27164/7101/64211/2260/5080 | 11 |
| GO:0050769 | BP | GO:0050769 | positive regulation of neurogenesis | 44/998 | 485/18866 | 353275 | 27093043 | 25549517 | 29993/147040/9699/6664/2290/118738/221914/22941/4825/5803/7077/9353/5468/2242/90527/8322/652/8837/2803/5649/4914/23011/57118/9181/443/1826/4821/2260/4803/84630/6009/4681/5080/4137/627/63950/22849/7516/127833/116/6857/4673/1453/5915 | 44 |
| GO:0032228 | BP | GO:0032228 | regulation of synaptic transmission, GABAergic | 8/998 | 35/18866 | 390778 | 29112967 | 27454364 | 57555/11188/627/4889/266743/6863/1269/8224 | 8 |
| GO:0110111 | BP | GO:0110111 | negative regulation of animal organ morphogenesis | 8/998 | 35/18866 | 390778 | 29112967 | 27454364 | 2303/1735/7068/652/55294/6909/340419/54880 | 8 |
| GO:0051703 | BP | GO:0051703 | intraspecies interaction between organisms | 10/998 | 53/18866 | 400479 | 2941544 | 27739605 | 2922/22941/94235/7101/5108/266743/9378/7349/342371/9369 | 10 |
| GO:0048705 | BP | GO:0048705 | skeletal system morphogenesis | 25/998 | 228/18866 | 457707 | 3315195 | 31263241 | 2303/3234/23314/6664/2778/8313/2253/4487/4088/652/4488/3227/4087/1739/2260/79977/5079/3221/162466/6913/55512/4323/3213/5915/157506 | 25 |
| GO:0050768 | BP | GO:0050768 | negative regulation of neurogenesis | 30/998 | 295/18866 | 478308 | 34169565 | 32222881 | 85458/6664/1735/11023/2290/152330/4825/9353/10363/6049/7068/6657/2672/9181/3670/7101/10509/4821/5780/6658/5108/5080/54910/627/10500/116/7431/23189/7070/7025 | 30 |
| GO:0048596 | BP | GO:0048596 | embryonic camera-type eye morphogenesis | 7/998 | 28/18866 | 504548 | 35557002 | 33531273 | 6664/2295/220/6670/6909/5080/51384 | 7 |
| GO:0007411 | BP | GO:0007411 | axon guidance | 29/998 | 284/18866 | 551613 | 3796383 | 35800982 | 11023/7204/2290/2253/152330/5015/9353/1945/1944/10716/2887/2668/5649/4914/399/284656/3670/10509/1796/1826/6711/8022/5080/54910/627/10500/10752/9378/9369 | 29 |
| GO:0071559 | BP | GO:0071559 | response to transforming growth factor beta | 27/998 | 258/18866 | 578164 | 3796383 | 35800982 | 463/51701/6664/8425/57178/23089/3622/857/3175/7316/4088/5654/128853/8837/56937/4087/51341/94031/203100/4092/1149/80310/5045/652991/4053/114990/2530 | 27 |
| GO:0035137 | BP | GO:0035137 | hindlimb morphogenesis | 8/998 | 37/18866 | 583594 | 3796383 | 35800982 | 2778/2253/4487/27164/652/4488/340419/5915 | 8 |
| GO:0048048 | BP | GO:0048048 | embryonic eye morphogenesis | 8/998 | 37/18866 | 583594 | 3796383 | 35800982 | 6664/2295/220/6670/6909/5080/51384/5915 | 8 |
| GO:0097485 | BP | GO:0097485 | neuron projection guidance | 29/998 | 285/18866 | 583928 | 3796383 | 35800982 | 11023/7204/2290/2253/152330/5015/9353/1945/1944/10716/2887/2668/5649/4914/399/284656/3670/10509/1796/1826/6711/8022/5080/54910/627/10500/10752/9378/9369 | 29 |
| GO:0001754 | BP | GO:0001754 | eye photoreceptor cell differentiation | 9/998 | 46/18866 | 586714 | 3796383 | 35800982 | 1735/23287/7068/57096/83552/1826/5080/7070/118427 | 9 |
| GO:2000678 | BP | GO:2000678 | negative regulation of transcription regulatory region DNA binding | 6/998 | 21/18866 | 589659 | 3796383 | 35800982 | 6664/4487/4488/55294/6717/7114 | 6 |
| GO:0006835 | BP | GO:0006835 | dicarboxylic acid transport | 14/998 | 98/18866 | 630135 | 39820557 | 37551929 | 56262/8541/6844/885/284111/2912/7200/627/2352/4889/2917/6857/9016/57030 | 14 |
| GO:0035116 | BP | GO:0035116 | embryonic hindlimb morphogenesis | 7/998 | 29/18866 | 635011 | 39820557 | 37551929 | 2778/2253/4487/652/4488/340419/5915 | 7 |
| GO:0002065 | BP | GO:0002065 | columnar/cuboidal epithelial cell differentiation | 16/998 | 121/18866 | 641405 | 39820557 | 37551929 | 6664/140628/2253/4825/7481/652/286262/10413/4821/2260/11078/6009/161497/286451/5080/5915 | 16 |
| GO:0003156 | BP | GO:0003156 | regulation of animal organ formation | 8/998 | 38/18866 | 705363 | 43276089 | 40810595 | 140628/2253/7481/652/2668/3227/3670/2260 | 8 |
| GO:0048663 | BP | GO:0048663 | neuron fate commitment | 11/998 | 67/18866 | 724515 | 43934246 | 41431256 | 23314/2290/4825/10716/219409/652/3670/4821/8022/5080/63950 | 11 |
| GO:0060395 | BP | GO:0060395 | SMAD protein signal transduction | 12/998 | 78/18866 | 772436 | 46301754 | 43663884 | 57178/121551/4088/652/353500/4087/6711/4092/9688/7431/25970/2657 | 12 |
| GO:0007216 | BP | GO:0007216 | G protein-coupled glutamate receptor signaling pathway | 5/998 | 15/18866 | 788858 | 4671434 | 44052964 | 2899/2914/2918/2912/2917 | 5 |
| GO:0043010 | BP | GO:0043010 | camera-type eye development | 32/998 | 332/18866 | 797234 | 4671434 | 44052964 | 2303/6664/1735/11023/2295/362/4088/30062/8322/220/5158/7068/57096/652/6670/6909/7101/22803/83552/1826/1739/79977/155051/4867/5080/10152/2823/7431/7070/51384/57030/157506 | 32 |
| GO:0051932 | BP | GO:0051932 | synaptic transmission, GABAergic | 9/998 | 48/18866 | 812403 | 47074269 | 44392387 | 57555/11188/2567/627/4889/266743/6863/1269/8224 | 9 |
| GO:0051961 | BP | GO:0051961 | negative regulation of nervous system development | 31/998 | 319/18866 | 834826 | 47483362 | 44778174 | 85458/6664/1735/11023/2290/152330/4825/9353/10363/6049/7068/27242/6657/2672/9181/3670/7101/10509/4821/5780/6658/5108/5080/54910/627/10500/116/7431/23189/7070/7025 | 31 |
| GO:0001662 | BP | GO:0001662 | behavioral fear response | 8/998 | 39/18866 | 846779 | 47483362 | 44778174 | 116372/3358/885/7101/4862/627/7349/116 | 8 |
| GO:0031076 | BP | GO:0031076 | embryonic camera-type eye development | 8/998 | 39/18866 | 846779 | 47483362 | 44778174 | 6664/2295/220/6670/6909/5080/51384/157506 | 8 |
| GO:0072001 | BP | GO:0072001 | renal system development | 29/998 | 292/18866 | 860509 | 4773992 | 45020115 | 2303/6664/2253/170690/4088/9353/7481/185/2650/652/8837/2668/3227/4087/5455/2018/10413/1739/2260/4092/80310/627/4609/64321/8772/4072/84445/5915/157506 | 29 |
| GO:0071560 | BP | GO:0071560 | cellular response to transforming growth factor beta stimulus | 26/998 | 252/18866 | 894399 | 49097813 | 46300648 | 51701/6664/8425/57178/23089/3622/857/3175/7316/4088/5654/128853/8837/56937/4087/51341/94031/203100/4092/1149/80310/5045/652991/4053/114990/2530 | 26 |
